# Parkinson’s disease mortality in the United States, 1999–2025: a national multiple-cause-of-death analysis with projections to 2030

**DOI:** 10.3389/fneur.2026.1934146

**Published:** 2026-10-01

**Authors:** Eshal Atif, Vishan Das, Mazhar Ali, Abdal Ahmad, Kaneez Fatima, Mohib Ali Hassan, Muhammad Mukhlis, Danyal Ahmad, Mohammad Dawar Zahid, Sadia Qazi, Muhammad Atif Mazhar, Mohammad Sunoqrot, Akef Obeidat

**Affiliations:** 1 College of Medicine, Alfaisal University, Riyadh, Saudi Arabia; 2 Liaquat University of Medical and Health Sciences, Jamshoro, Sindh, Pakistan; 3 Khyber Medical College, Peshawar, Pakistan; 4 Department of Medicine, Peshawar Medical College, Riphah International University (Peshawar Campus), Peshawar, Pakistan; 5 CMH Institute of Medical Sciences, Multan, Pakistan; 6 Rawal Institute of Health Sciences, Islamabad, Pakistan; 7 Ayub Medical College, Abbottabad, Pakistan; 8 Peshawar Institute of Cardiology, Peshawar, Pakistan; 9 Medical College, Aga Khan University, Karachi, Pakistan; 10 Department of Anatomy, College of Medicine, Alfaisal University, Riyadh, Saudi Arabia; 11 Department of Neurology, Johns Hopkins University School of Medicine, Baltimore, MD, United States

**Keywords:** forecasting, health disparities, joinpoint regression, long-term care, mortality, palliative care, Parkinson’s disease

## Abstract

**Background:**

Parkinson’s disease (PD) is the second most common neurodegenerative disorder in the United States and a growing driver of late-life disability and death. We characterized U.S. PD-related mortality from 1999 to 2025 and projected it to 2030.

**Methods:**

In this serial cross-sectional analysis of CDC WONDER Multiple Cause of Death data, PD-related deaths in adults aged ≥45 years were identified using ICD-10 code G20 recorded anywhere on the death certificate. Age-adjusted mortality rates (AAMRs) per 100,000 were decomposed by joinpoint regression and projected to 2030 using a log-scale ensemble weighted by rolling-origin back-test error, stratified by sex, age, race and ethnicity, census region, and degree of urbanization.

**Results:**

Among 1,149,731 PD-related deaths (59.8% men), the overall AAMR was 32.74 in 1999 and 41.03 (95% CI 40.70–41.35) in 2025, but the full-window trend was not significant (average annual percent change 0.56%; −0.10 to 1.22; *p* = 0.099). A steep 2014–2021 rise (annual percent change 4.70%; *p* < 0.001) was followed by a non-significant decline, negative in 13 of the 14 strata observed through 2025. COVID-19 was co-listed on 10.15% of PD-mentioned deaths in 2020 and 0.79% in 2025, accounting for 58% of the 2019–2020 rise and 55% of the 2021–2024 decline; excluding these deaths the series was close to flat (39.88 in 2020; 40.71 in 2025). The projected 2030 AAMR was 41.73 (95% prediction interval 33.97–51.27), essentially unchanged from 2025; however, deaths are projected to rise from 62,162 to 70,825, with approximately 87% of the increase attributable to population aging. Restricting to PD as the underlying cause gave a steeper increase (+86.2% versus +25.3%), arguing against ascertainment drift. Between 1999–2007 and 2017–2025, nursing home deaths fell from 49.24 to 35.48%, while home deaths rose from 19.37 to 34.93% and hospice deaths from 0.88 to 8.25%.

**Conclusion:**

Age-adjusted PD-related mortality did not change significantly between 1999 and 2025. Most of the post-2021 decline reflects the unwinding of COVID-19 co-listing rather than an improvement in underlying mortality, a mechanism that is now nearly exhausted. Projections indicate a plateau in the standardized rate, while absolute deaths rise with population aging, and end-of-life care has shifted from institutions to homes.

## 1 Introduction

Parkinson’s disease (PD) is a progressive neurodegenerative disorder clinically defined by bradykinesia, rest tremor, rigidity, and postural instability, along with autonomic, neuropsychiatric, and cognitive features. As a leading cause of age-related neurological disability, PD is an important public health concern as the U.S. population ages and survival with chronic neurodegenerative diseases lengthens (1–4). Mortality surveillance retains particular value because PD deaths typically mark the terminus of a protracted course of progressive disability, institutionalization, caregiver dependence, and escalating palliative care need (5–7).

Aggregate national statistics obscure the substantial heterogeneity in the recorded burden of PD. Prior studies have documented consistent sex differences, with higher incidence, prevalence, and mortality rates among men than among women (8). Recorded PD mortality is typically highest among non-Hispanic White individuals, whereas lower recorded rates among Black, Asian, Hispanic, American Indian, Alaska Native, Native Hawaiian, and Pacific Islander populations may partly reflect diagnostic delay, under-ascertainment, differential access to neurologic care, or competing mortality, rather than a genuinely lower burden of disease (9, 10). Geographic variation is similarly relevant for health-system planning: the PD burden has been linked to regional and rural residence, environmental and occupational exposures, disparities in healthcare access, long-term care capacity, and the uneven distribution of movement disorder specialists (11, 12). Non-metropolitan communities may face compounding barriers, including delayed diagnosis, transportation constraints, limited rehabilitation infrastructure, sparse specialist coverage, and weaker home and facility-based support.

Several recent U.S. analyses have used CDC WONDER data to describe PD mortality trends and disparities, including national analyses with windows ending between 2019 and 2023, machine learning-based forecasting, and race-stratified joinpoint analyses (12–16). These have consistently corroborated higher recorded mortality among men and White individuals but have generally been constrained by shorter observation windows that end before the post-2020 inflection or by coarser subgroup resolution. The present analysis was designed to extend that record: we combined joinpoint decomposition of a 27-year window with subgroup-specific ensemble forecasting across sex, age, race and ethnicity, census region, and urbanization, together with descriptive state-level analysis, and linked these trends to place-of-death distributions with direct relevance to long-term and palliative care.

## 2 Methods

### 2.1 Study design and data source

We conducted a serial cross-sectional analysis of national death certificate data using the CDC Wide-ranging Online Data for Epidemiologic Research (CDC WONDER) Multiple Cause of Death public-use database, which compiles death certificates filed in all 50 states and the District of Columbia and records one underlying cause, up to 20 contributing causes, and standard demographic variables (17).

PD-related deaths were ascertained using the ICD-10 code G20 (18). Deaths were included when G20 appeared anywhere on the death certificate, whether as the underlying or contributing cause, an approach that captures PD-related mortality more broadly than analyses restricted to PD as the sole underlying cause. Secondary parkinsonism (G21) was deliberately excluded because it denotes drug-induced, post-infectious, and vascular syndromes of distinct etiology and certification practice. We note that the National Center for Health Statistics defines PD mortality using G20 and G21 together; therefore, our estimates are not directly comparable to that reporting convention, and we report a G20 + G21 sensitivity analysis.

The analytic window extended from January 1, 1999, to December 31, 2025. The analysis relied on multiple CDC WONDER data extractions, each of which is reported here with its extraction date because the records in the 2025 file were still being accrued. The primary extraction, conducted on June 26, 2026, provided all counts and rates used in the main analysis. It included the Multiple Cause of Death 1999–2020 file (bridged race) and Multiple Cause of Death 2018–2025 file (single race).

Subsequent sensitivity extractions were performed in August 2026. These comprised a re-query of the single-race 2025 counts and the COVID-19 (U07.1) co-listing series on August 7, 2026, and the direct Hispanic-origin series on August 8, 2026; during the same round, we also extracted the underlying cause and G20 + G21 case-definition series. This re-query was substantive, rather than solely administrative. Between June 26 and August 7, 2026, the recorded 2025 counts increased across all four non-Hispanic race groups, with an additional 102 deaths in total, whereas the 2024 counts, which were final, remained identical to the original extraction.

Data for 1999–2024 are final, and those for 2025 are provisional. Because this study used publicly available, deidentified data, institutional review board approval and informed consent were not required. Reporting followed the STROBE guidelines for observational studies (19), and the completed checklist is provided in Appendix A.

### 2.2 Population and stratification

The analysis included all U.S. PD-related deaths in the database during the study window; because reported counts below age 45 were negligible, the analytic population comprised adults aged ≥45 years. Age was stratified into 45–64 and ≥65 years. Sex was classified as male or female.

Race and ethnicity were derived from a single cross-classified extraction of the Race and Hispanic Origin variables, which were recorded independently in the Multiple Cause of Death files and were grouped into five categories: non-Hispanic White, non-Hispanic Black or African American, Hispanic or Latino, non-Hispanic American Indian or Alaska Native, and non-Hispanic Asian or Pacific Islander. These categories are mutually exclusive but do not exhaust the analytic population: non-Hispanic decedents recorded as more than one race (*n* = 859, single-race years only, with no bridged-race counterpart) and decedents whose Hispanic origin was not stated (*n* = 1,910) fall outside the Hispanic or Latino stratum and were not redistributed. One qualification applies to the earlier era and is stated here because it bears on how the supplementary tables should be interpreted. In the bridged-race years 1999–2020, the four non-Hispanic race series sum exactly, in all 22 years, to the annual total minus Hispanic or Latino deaths, so the 1,400 deaths of unstated origin recorded in that period (0.12% of the analytic population) are contained within those four series rather than excluded from them; only the 510 recorded in 2021–2025 fall outside all five categories. These labels are used consistently throughout the manuscript, tables, and figures.

The 1999–2025 series was constructed from two separate CDC WONDER extractions because the database transitioned from bridged race to single-race coding during the study period. Deaths for 1999–2020 were obtained from the Multiple Cause of Death 1999–2020 file (bridged race), and deaths for 2021–2025 were obtained from the Multiple Cause of Death 2018–2025 (Single Race) file, using the Single Race 6 classification restricted to non-Hispanic or Latino individuals. For single-race years, the Asian and Native Hawaiian or Other Pacific Islander categories were combined to reconstruct the Asian or Pacific Islander category used in the bridged-race era. We confirmed that these combined counts reproduced the reported totals for 2021–2024. For 2025, the reconstructed total exceeded the reported count by 10 deaths because the single-race detail was derived from the August 7, 2026, re-query, whereas the race counts were from June 26, 2026 (Section 2.1).

Two consequences of this construction should be noted. First, the combined Asian or Pacific Islander category averages a large and stable Asian series (1,400–2,078 deaths annually) with a very small and volatile Native Hawaiian or Other Pacific Islander series (22–42 deaths annually, whose age-adjusted rate ranges from 12.24 to 26.73). The combined estimate is therefore dominated by the Asian component and should not be read as informative about Native Hawaiian or Pacific Islander populations. Second, the single-race files include a “More than one race” category with no bridged-race counterpart (164–179 deaths annually in 2021–2025).

Hispanic or Latino deaths were obtained by direct query on the Hispanic Origin field across all races, rather than by subtraction, because subtraction assigns records of unstated origin to the Hispanic category. Records for which Hispanic origin was not stated were excluded from the Hispanic or Latino stratum; these accounted for 1,910 deaths across 1999–2025, or 0.166% of the analytic population. Their treatment in the four non-Hispanic race series differed by era, as described above.

Place of death followed the standard CDC WONDER categories. County-level urbanization used the 2013 National Center for Health Statistics Urban–Rural Classification Scheme, collapsed into metropolitan and non-metropolitan (20). Census regions followed U.S. Census Bureau definitions. State-level analyses were conducted separately for 1999–2020 and 2021–2025 because post-2020 CDC WONDER reliability and suppression rules preclude continuous state-level trend modeling across the full window.

### 2.3 Outcome measures

The primary outcome was the annual age-adjusted mortality rate (AAMR) per 100,000, standardized to the 2000 U.S. Standard Population. Secondary analyses reported AAMRs for each stratum. Place-of-death frequencies were reported as proportions of total PD-related deaths, both pooled across the window and by period (1999–2007, 2008–2016, 2017–2025), because national end-of-life care patterns changed substantially over 27 years. Where period-level summary rates are quoted, we report pooled period AAMRs.

To quantify the contribution of COVID-19 to the late-period surge, we extracted deaths in which G20 and U07.1 were both recorded, using the two-box AND structure of the CDC WONDER Provisional Mortality Statistics file (2018 onward) (21) over the same age range, and expressed these as a proportion of all PD-mentioned deaths per year. Because age-adjusted rates are additive across mutually exclusive subsets of deaths sharing the same population denominator, a PD-excluding-COVID series was obtained by subtracting the co-listed age-adjusted rate from the all-PD age-adjusted rate.

Observed disparities were quantified as rate ratios between contrasting strata at the start and end of the window and in 2019, where a pre-pandemic comparison is informative. Because strata were modeled independently, no projected disparity measures are reported (see Section 2.5).

### 2.4 Trend analysis

Secular trends from 1999 to 2025 were assessed using Joinpoint Regression Program version 5.4.0 (22). Annual AAMRs were modeled on a log-linear scale. We estimated the annual percent change (APC) for each segment and the average annual percent change (AAPC) across the full window, each with 95% confidence intervals (CIs). Inflection points were identified using the Monte Carlo permutation method (23), and the weighted Bayesian Information Criterion guided model selection. An APC or AAPC was classified as increasing or decreasing when the slope differed significantly from zero at *α* = 0.05 (two-sided).

CDC WONDER suppresses cells based on fewer than 10 deaths and flags rates based on fewer than 20 deaths as unreliable. No annual national or stratum-level cell in the demographic, regional, or urbanization series fell below these thresholds, so no suppression handling was required for the joinpoint fits; suppression affected only state-by-year cells, which is why state analyses are reported as two pooled windows rather than annual series.

Sixteen strata and 51 state estimates were evaluated. No adjustment was made for multiplicity, and stratum-level *p*-values should therefore be read as descriptive rather than as confirmatory hypothesis tests.

### 2.5 Forecasting analysis

Projections were generated for every stratum using an ensemble of three models: Holt linear-trend exponential smoothing, additive damped-trend exponential smoothing, and an automatically selected ARIMA model implemented in Python with the Statsmodels package. Several features of the specification are material and explicitly stated.

#### 2.5.1 Scale

All models were fitted to the natural logarithm of the AAMR, and point estimates and interval bounds were exponentiated back to the rate scale. Log-scale fitting guarantees strictly positive, asymmetric prediction intervals; an additive model on the rate scale admits negative mortality rates and produces symmetric intervals that are not credible at long horizons.

#### 2.5.2 ARIMA selection

Orders were chosen by AICc grid search over *p* ≤ 3, d ≤ 2, q ≤ 3 with stationarity and invertibility enforced, and with a constant required at d = 0 so that the process reverts to the level of the series. Relaxing the stationarity constraint permits an explosive autoregressive root, which extrapolates by compounding rather than by any estimated trend; we verified that such models were both less accurate in back-testing and qualitatively different in direction, and they were excluded.

#### 2.5.3 Forecast origin

Projections are generated from the fitted model rather than by carrying the last observation forward, so the first projected value does not in general equal the terminal observed rate. Where the selected specification is differenced (*d* = 1), the forecast begins at approximately the last observation; where it is stationary with a constant (*d* = 0), the forecast begins at a fitted value that has already reverted partway toward the estimated mean. The largest such step in our results is in the American Indian or Alaska Native stratum, whose selected model is a moving-average process about a constant and whose 2026 estimate is 23.19 against an observed 21.69 in 2025.

#### 2.5.4 Weighting

Members were combined by weights inversely proportional to each model’s rolling-origin back-test error, averaged over forecast horizons of 1 to 5 years. Back-testing used rolling origins with a minimum training window of 12 years. The two pandemic years, 2020 and 2021, were retained in training but excluded as back-test targets, on the ground that reproducing an exogenous shock is not the standard against which a secular trend model should be calibrated. This choice is material to the calibration we report: it removes the 2 years that any earlier training window would have missed by the widest margin, so every mean absolute percentage error and bias figure in Section 3.8 is more favorable than it would be if the pandemic years were included. Weights are non-negative and sum to one. No weight was capped, extrapolated beyond the unit interval, or set negative. Fitted weights and selected ARIMA orders are reported for every stratum. Prediction intervals combine within-model forecast variance and between-model dispersion on the log scale.

#### 2.5.5 Horizon

The forecast horizon was set to 2030 on empirical grounds. With 27 annual observations, rolling-origin validation is feasible only to about 10 years ahead, and even a ten-year horizon rests on four validation origins. The ensemble back-test error for the overall series rose monotonically with the horizon, from 4.2% at 1 year to 13.4% at 5 years and 24.0% at 10 years. A 25-year projection to 2050 lies two-and-a-half times beyond the longest horizon that these data can validate at all, and we therefore do not report one.

#### 2.5.6 Reportability rule

A single pre-specified quantitative rule was applied uniformly to every stratum. A projection is reported only if (i) the series comprises at least 20 annual observations extending to the most recent data year and (ii) the rolling-origin ensemble back-test error at the five-year horizon is ≤20%, the conventional threshold for good forecasting accuracy (24). Strata failing either criterion are reported as “not reported” (NR) with their observed trend given instead. Fourteen of sixteen strata met both criteria. The two urbanization strata failed criterion (i). Urbanization-specific forecasts are not extended beyond 2020 because the available NCHS urbanization data end in that year, and because the terminal observation coincides with the COVID-19 pandemic period, which makes long-range extrapolation from that endpoint unstable and potentially misleading; every candidate model extrapolated the pandemic-year peak to implausible values. The urbanization analysis is therefore restricted to observed 1999–2020 temporal trends, and no post-2020 projections are presented.

#### 2.5.7 Independent replication

As a check on the ensemble, we implemented a separate ARIMA analysis using different software without reference to the ensemble specification. Only 2030 point estimates were compared because that implementation generated intervals on the rate rather than the log scale.

#### 2.5.8 Specifications

Two specifications are reported side by side. The primary specification uses the full 1999–2025 series, which includes the observed post-2021 decline. A sensitivity specification is anchored on the pre-pandemic 1999–2019 window and represents continuation of the pre-pandemic secular trend. The full-data specification is primary because the post-2021 observations now exist and are informative about the current direction; the pre-pandemic anchor is retained because it isolates the secular trend from pandemic disruption.

Because each stratum was modeled independently, subgroup projections are not constrained to aggregate to the national projection and are presented as stratum-specific directional estimates. Projected between-group differences accordingly carry no valid joint uncertainty and are not interpreted as projected disparities.

### 2.6 Projected death counts

Because age-adjusted rates remove population aging by construction, they cannot by themselves indicate the number of deaths a health system must plan for. We therefore converted projected rates into projected counts using the U. S. Census Bureau 2023 National Population Projections (main series), the first projection series based on the 2020 Census, with a base population of July 1, 2022 (25).

For each age band b (45–64 and ≥65 years), the observed 2025 band rate was computed as deaths divided by the projected 2025 population, scaled forward by the ratio of the projected to the observed 2025 age-adjusted rate for that band, and applied to the projected population for each year:


$$\begin{array}{c} D(b,t)=[D(b,2025)/P(b,2025)]\\ \times [\text{AAMR}(b,t)/\text{AAMR}(b,2025)]\times P(b,t) \end{array}$$

Prediction intervals for counts were obtained by carrying the rate interval bounds through the same calculation. Census projections were used for the base-year denominator as well as the forecast years so that the ratio is internally consistent; these differ slightly from the postcensal estimates CDC WONDER uses for its own denominators.

To separate the demographic contribution from the epidemiological one, we additionally computed a constant-rate scenario in which the age-adjusted rate is held at its observed 2025 value and only the population is allowed to change. The difference between the two scenarios isolates how much of the projected change in deaths is attributable to population aging alone. This calculation assumes that the age composition within each band is stable, so that the age-standardized rate ratio transfers to the band’s crude rate; this assumption is stated in the Limitations.

### 2.7 Software

Data preprocessing, model fitting, back-testing, and visualization were performed in Python (statsmodels); joinpoint analyses used the Joinpoint Regression Program. All tests were two-sided, with *p* < 0.05 considered significant.

## 3 Results

### 3.1 Overall burden and place of death

Between 1999 and 2025, 1,149,731 PD-related deaths were recorded (Table 1). Males accounted for 687,809 deaths (59.8%) and females for 461,922 (40.2%).

**Table 1:** Parkinson’s disease-related deaths and age-adjusted mortality rates (AAMR) per 100,000 in U.S. adults aged ≥45 years, 1999–2025, with projections to 2030.

Subgroup	Deaths, no.	AAMR 1999 (95% CI)	AAMR 2025 (95% CI)	AAPC, % (95% CI); *p*	Projected AAMR 2030 (95% PI)	Sensitivity: pre-pandemic anchor (95% PI)
Overall	1,149,731	32.74 (32.38–33.11)	41.03 (40.70–41.35)	0.56 (−0.10 to 1.22); 0.099	41.73 (33.97 to 51.27)	38.27 (29.59 to 49.50)
Sex						
Female	461,922	22.48 (22.10–22.86)	26.37 (26.03–26.72)	0.33 (−0.25 to 0.92); 0.267	26.52 (21.30 to 33.01)	23.54 (19.83 to 27.95)
Male	687,809	50.85 (50.08–51.62)	61.01 (60.40–61.63)	0.40 (−0.25 to 1.05); 0.225	61.53 (50.37 to 75.16)	60.08 (40.60 to 88.90)
Age group, years						
45–64	29,402	0.95 (0.87–1.02)	1.52 (1.44–1.60)	**1.84 (0.34 to 3.35); 0.016** ^b^	1.54 (1.12 to 2.14)	1.48 (1.00 to 2.21)
≥65	1,120,329	88.62 (87.62–89.61)	110.45 (109.57–111.34)	**0.60 (0.08 to 1.12); 0.024** ^b^	112.27 (91.40 to 137.91)	109.72 (72.50 to 166.05)
Race and ethnicity						
White, non-Hispanic	1,006,092	34.97 (34.56–35.37)	45.91 (45.51–46.30)	**0.76 (0.13 to 1.40); 0.018** ^b^	47.00 (38.30 to 57.68)	42.30 (32.71 to 54.70)
Hispanic or Latino	59,467	22.28 (20.75–23.80)	28.61 (27.73–29.52)	0.64 (−0.67 to 1.98); 0.339	29.28 (23.43 to 36.60)	29.81 (23.67 to 37.54)
American Indian or Alaska Native, non-Hispanic	3,641	23.68 (18.42–29.97)	21.69 (18.81–24.93)	−0.94 (−2.33 to 0.47); 0.192	23.47 (19.28 to 28.56)	23.82 (20.03 to 28.34)
Asian or Pacific Islander, non-Hispanic	28,194	21.14 (18.94–23.34)	25.29 (24.22–26.40)	0.37 (−0.40 to 1.15); 0.343	25.17 (19.67 to 32.21)	30.06 (14.48 to 62.42)
Black or African American, non-Hispanic	52,486	16.81 (15.87–17.76)	23.24 (22.43–24.07)	**0.93 (0.19 to 1.68); 0.014** ^b^	23.50 (17.68 to 31.24)	21.68 (16.16 to 29.07)
Census region						
Northeast	204,753	30.19 (29.44–30.95)	35.38 (34.69–36.08)	0.57 (−0.08 to 1.23); 0.083	34.08 (27.93 to 41.59)	33.40 (23.66 to 47.14)
Midwest	280,525	36.46 (35.68–37.24)	44.39 (43.66–45.13)	0.52 (−0.06 to 1.10); 0.078	44.85 (35.86 to 56.09)	41.39 (32.17 to 53.24)
South	408,434	29.55 (28.96–30.14)	42.72 (42.19–43.27)	**1.06 (0.41 to 1.72); 0.001** ^b^	46.74 (37.11 to 58.86)	39.06 (28.37 to 53.78)
West	256,019	36.73 (35.85–37.60)	39.71 (39.04–40.39)	0.07 (−0.47 to 0.61); 0.800	38.96 (32.63 to 46.51)	37.83 (31.59 to 45.29)
Urbanization (1999–2020)^a^						
Metropolitan	697,136	32.99 (32.58–33.40)	43.93 (43.55–44.31)^a^	**0.85 (0.27 to 1.44); 0.004** ^b^	NR	NR
Non-metropolitan	154,676	31.71 (30.90–32.52)	46.43 (45.57–47.30)^a^	**1.28 (0.75 to 1.82); <0.001** ^b^	NR	NR

AAMR, age-adjusted mortality rate per 100,000 (2000 U.S. standard); AAPC, average annual percent change; CI, confidence interval; PI, prediction interval; NR, not reported. Deaths are 1999–2025 totals; bold AAPC denotes *p* < 0.05.

a Urbanization data ended in 2020; those rows show 2020 values and the 1999–2020 AAPC. Projections: log-scale ensemble of Holt, damped-trend, and ARIMA weighted by rolling-origin back-test error, fitted to 1999–2025 (primary) or 1999–2019 (sensitivity). The strata were modeled independently and were not additive. Shown only where a stratum has ≥20 observations to 2025 and five-year back-test error ≤20%, which both urbanization strata fail (NR).

b Bold AAPC values indicate trends that are statistically significant at *p* < 0.05.

Pooled across the full window, most deaths occurred in nursing homes or long-term care facilities (41.11%), followed by decedents’ homes (28.58%) and inpatient medical facilities (16.26%), with hospice facilities accounting for 5.71%. These pooled figures conceal a substantial redistribution over 27 years, and we therefore report place of death by period (Supplementary Table S1).

Between 1999–2007 and 2017–2025, the share of PD-related deaths occurring in nursing homes or long-term care fell from 49.24 to 35.48%, and the share occurring in inpatient medical facilities fell from 22.68 to 12.93%. Over the same interval the share occurring in decedents’ homes rose from 19.37 to 34.93%, and the share occurring in hospice facilities rose from 0.88 to 8.25%, a more than ninefold increase. In the most recent period, deaths at home (34.93%) and deaths in nursing homes or long-term care (35.48%) are of almost equal magnitude.

### 3.2 Overall trend and projection

The overall AAMR was 32.74 per 100,000 (95% CI 32.38–33.11) in 1999 and 41.03 (40.70–41.35) in 2025, the latter based on provisional data; the full annual series is given in Supplementary Table S2. Across the full window the trend was not statistically significant (AAPC 0.56%; 95% CI −0.10 to 1.22; *p* = 0.099) (Figure 1).

**Figure 1:**
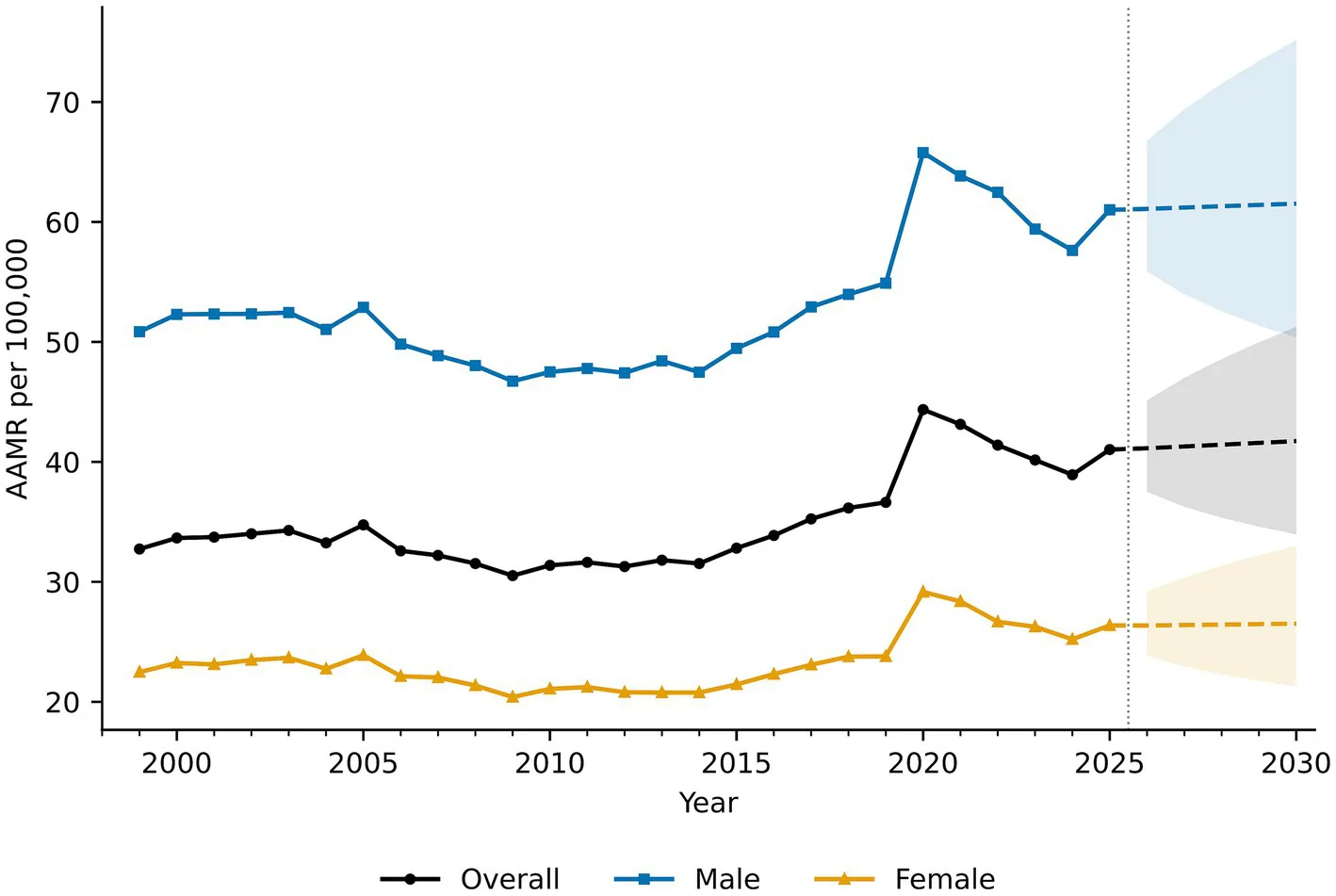
Age-adjusted Parkinson’s disease-related mortality overall and by sex, United States, 1999–2030. Solid lines with markers represent observed AAMRs per 100,000 from 1999 through 2025; dashed lines represent ensemble projections from 2026 through 2030; shaded bands represent 95% prediction intervals from the log-scale ensemble. The dotted vertical line marks the boundary between observed and projected data. Rates were standardized to the 2000 U.S. standard population. AAMR, age-adjusted mortality rate.

This flat net trend masked three phases. Joinpoint analysis identified a significant decline from 1999 to 2014 (APC −0.65%; 95% CI −1.16 to −0.14; *p* = 0.015), a steep and highly significant increase from 2014 to 2021 (APC 4.70%; 2.95 to 6.48; *p* < 0.001), and a non-significant decline from 2021 to 2025 (APC −1.96%; −4.78 to 0.94; *p* = 0.171). Four of the last five observed years declined. The 2014–2021 surge is the dominant feature of the record; the full-window average is not.

The ensemble projected an overall AAMR of 41.73 per 100,000 in 2030 (95% prediction interval 33.97–51.27), essentially unchanged from the 2025 observed value of 41.03. The pre-pandemic-anchored sensitivity specification projected 38.27 (29.59–49.50). Both specifications indicate a near-term plateau rather than continued growth, and their intervals overlap substantially. Segment-level APCs for every stratum are provided in Supplementary Table S3; full projected series with prediction intervals are in Supplementary Table S4.

### 3.3 Sex

Both sexes were at higher AAMRs in 2025 than in 1999, but neither full-window trend reached significance. Female AAMRs moved from 22.48 to 26.37 (AAPC 0.33%; 95% CI −0.25 to 0.92; *p* = 0.267) and male AAMRs from 50.85 to 61.01 (AAPC 0.40%; −0.25 to 1.05; *p* = 0.225). Both sexes showed the same three-phase pattern, with terminal segments declining (female 2020–2025 APC −1.41%, *p* = 0.085; male 2021–2025 APC −1.98%, *p* = 0.151).

The male excess was large and stable: the male-to-female rate ratio was 2.26 in 1999, 2.31 in 2019, and 2.31 in 2025. Projections estimate female AAMRs of 26.52 (95% PI 21.30–33.01) and male AAMRs of 61.53 (50.37–75.16) in 2030 (Figure 1).

### 3.4 Age

PD-related mortality was concentrated among adults aged ≥65 years, whose AAMR was roughly seventy-fold that of adults aged 45–64. In the 45–64 stratum, AAMRs rose from 0.95 to 1.52 (AAPC 1.84%; 95% CI 0.34 to 3.35; *p* = 0.016); in the ≥65 stratum, from 88.62 to 110.45 (AAPC 0.60%; 0.08 to 1.12; *p* = 0.024). The 45–64 stratum declined significantly in the terminal segment (APC −2.71%, *p* = 0.045), and the ≥65 terminal segment was also negative but did not reach significance (APC −1.12%, *p* = 0.114).

Projections estimate 1.54 (95% PI 1.12–2.14) for adults aged 45–64 and 112.27 (91.40–137.91) for adults aged ≥65 in 2030 (Figure 2; Supplementary Table S5).

**Figure 2:**
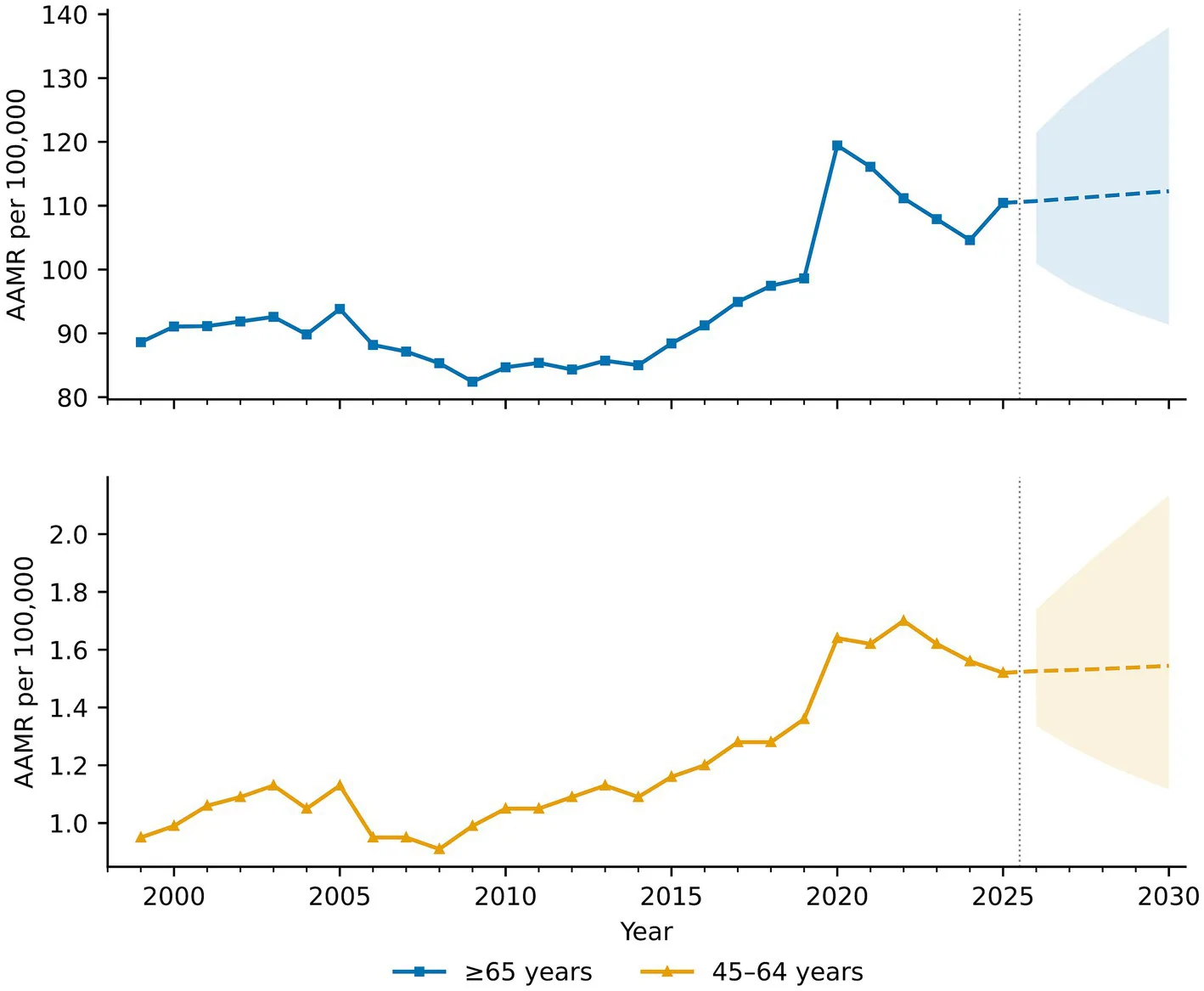
Age-adjusted Parkinson’s disease-related mortality by age group, United States, 1999–2030. Panels are plotted on separate vertical scales rather than sharing a dual axis, because the two age strata differ by approximately seventyfold and cannot be read reliably on a common scale. Solid lines with markers represent observed AAMRs; dashed lines represent ensemble projections through 2030; shaded bands represent 95% prediction intervals. AAMR, age-adjusted mortality rate.

### 3.5 Race and ethnicity

Non-Hispanic White individuals had the highest AAMR in every year of the window (Supplementary Table S6). The ordering of the remaining groups was not stable: Hispanic or Latino individuals had the second-highest rate in 19 of the 27 years, and non-Hispanic American Indian or Alaska Native individuals had the second-highest rate in the other eight. The American Indian or Alaska Native rate fell below the non-Hispanic Black or African American rate in the final 3 years of the study. Non-Hispanic White AAMRs moved from 34.97 to 45.91 (AAPC 0.76%; 95% CI 0.13 to 1.40; *p* = 0.018) and non-Hispanic Black or African American AAMRs from 16.81 to 23.24 (AAPC 0.93%; 0.19 to 1.68; *p* = 0.014).

These two AAPC estimates have heavily overlapping confidence intervals and were not formally compared; we therefore describe the Black or African American AAPC as the highest point estimate among racial and ethnic groups rather than as a significantly larger increase. Hispanic or Latino AAMRs moved from 22.28 to 28.61 on direct query (AAPC 0.64%; 95% CI −0.67 to 1.98; *p* = 0.339; Supplementary Table S7, which also reports records with Hispanic origin not stated); Hispanic or Latino, Asian or Pacific Islander, and American Indian or Alaska Native full-window trends were not statistically significant, and each of these three strata declined significantly in its terminal segment (Hispanic or Latino 2020–2025 APC −3.76%, *p* = 0.002; American Indian or Alaska Native 2020–2025 APC −6.18%, *p* = 0.006; Asian or Pacific Islander 2021–2025 APC −3.21%, *p* = 0.037).

Under the uniform reportability rule, all five strata met both criteria at the 2030 horizon, and projections were reported for each (Table 1; Figure 3; Supplementary Table S6).

**Figure 3:**
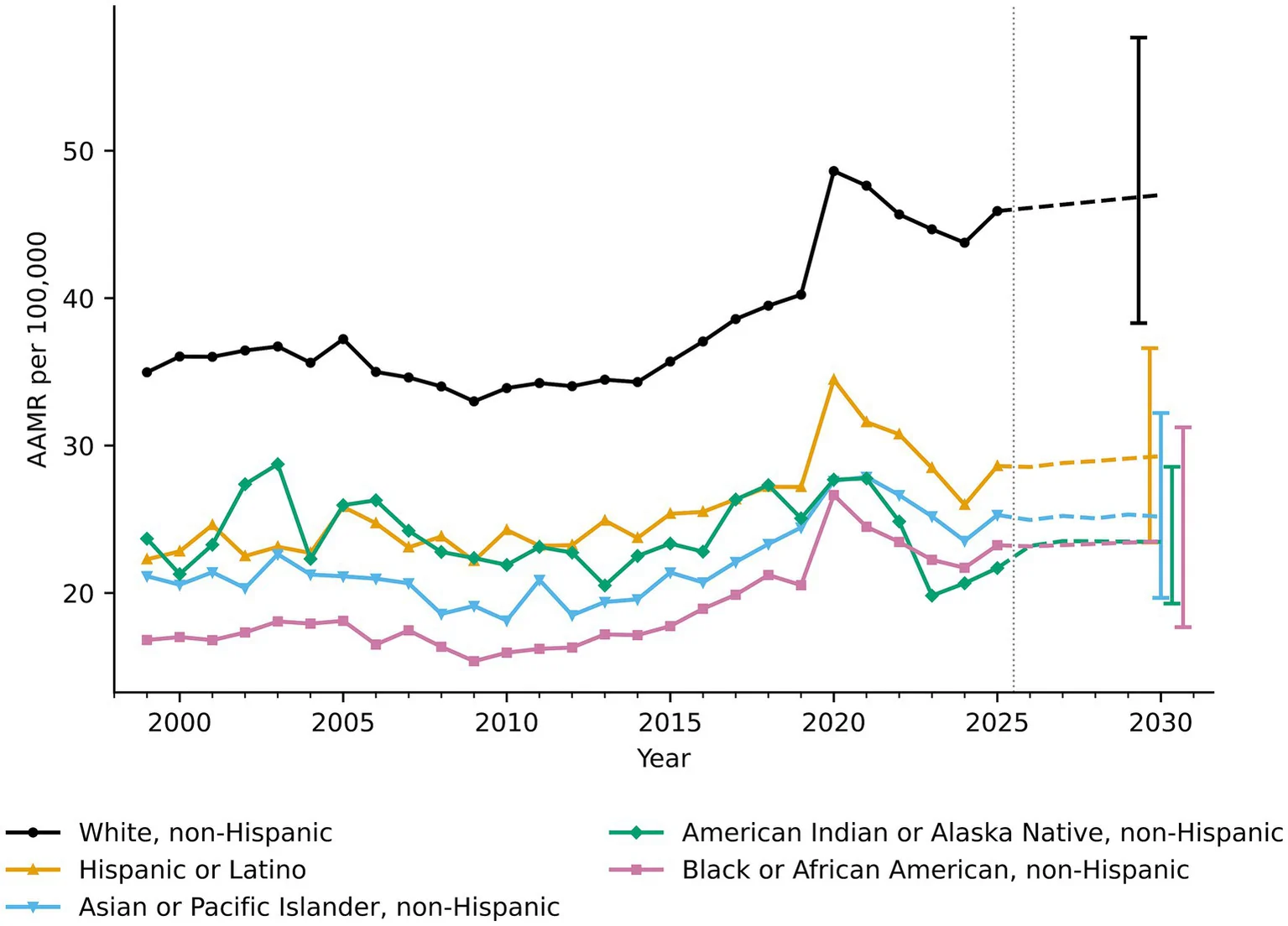
Age-adjusted Parkinson’s disease-related mortality by race and ethnicity, United States, 1999–2030. Solid lines with markers represent observed AAMRs from 1999 through 2025; dashed lines represent ensemble projections from 2026 through 2030. Vertical bars at 2030 represent 95% prediction intervals, offset horizontally to avoid overlap; intervals are shown as bars at the horizon year rather than as continuous shaded bands, because five overlapping bands occlude one another and cannot be read reliably. All five strata met the pre-specified reportability criteria (≥20 annual observations extending to 2025 and five-year back-test error ≤20%) and are therefore projected. Categories are mutually exclusive and are non-Hispanic except Hispanic or Latino. AAMR, age-adjusted mortality rate.

Observed disparities narrowed modestly for one contrast and widened for another: the non-Hispanic White to non-Hispanic Black or African American rate ratio was 2.08 in 1999 and 1.98 in 2025, whereas the non-Hispanic White to Hispanic or Latino ratio was 1.57 in 1999 and 1.60 in 2025. The Asian and Native Hawaiian or Other Pacific Islander components of the combined category are reported separately in Supplementary Table S8: the Asian component contributes 1,400–2,078 deaths annually at a stable rate, while the Native Hawaiian or Other Pacific Islander component contributes 22–42 deaths annually at a rate ranging from 12.24 to 26.73, so the combined estimate is dominated by the Asian component.

### 3.6 Urbanization

Both metropolitan and non-metropolitan areas showed significant increases across 1999–2020. Metropolitan AAMRs rose from 32.99 to 43.93 (AAPC 0.85%; 95% CI 0.27 to 1.44; *p* = 0.004) and non-metropolitan AAMRs from 31.71 to 46.43 (AAPC 1.28%; 0.75 to 1.82; *p* < 0.001).

Non-metropolitan AAMRs first exceeded metropolitan AAMRs in 2003 (34.47 vs. 34.27) and were higher in 16 of the 22 observed years; metropolitan rates were higher in 1999–2002, 2004, and 2014. For most of the first two decades the two series were closely superimposed, with differences well within their confidence intervals. What changed in the late period is the size of the gap rather than the ordering: the non-metropolitan excess widened from 0.04 per 100,000 in 2013 to 2.39 in 2019 and 2.50 in 2020 (46.43 vs. 43.93), and the non-metropolitan-to-metropolitan rate ratio moved from 0.96 in 1999 to 1.07 in 2019 (Figure 4; Supplementary Table S9).

**Figure 4:**
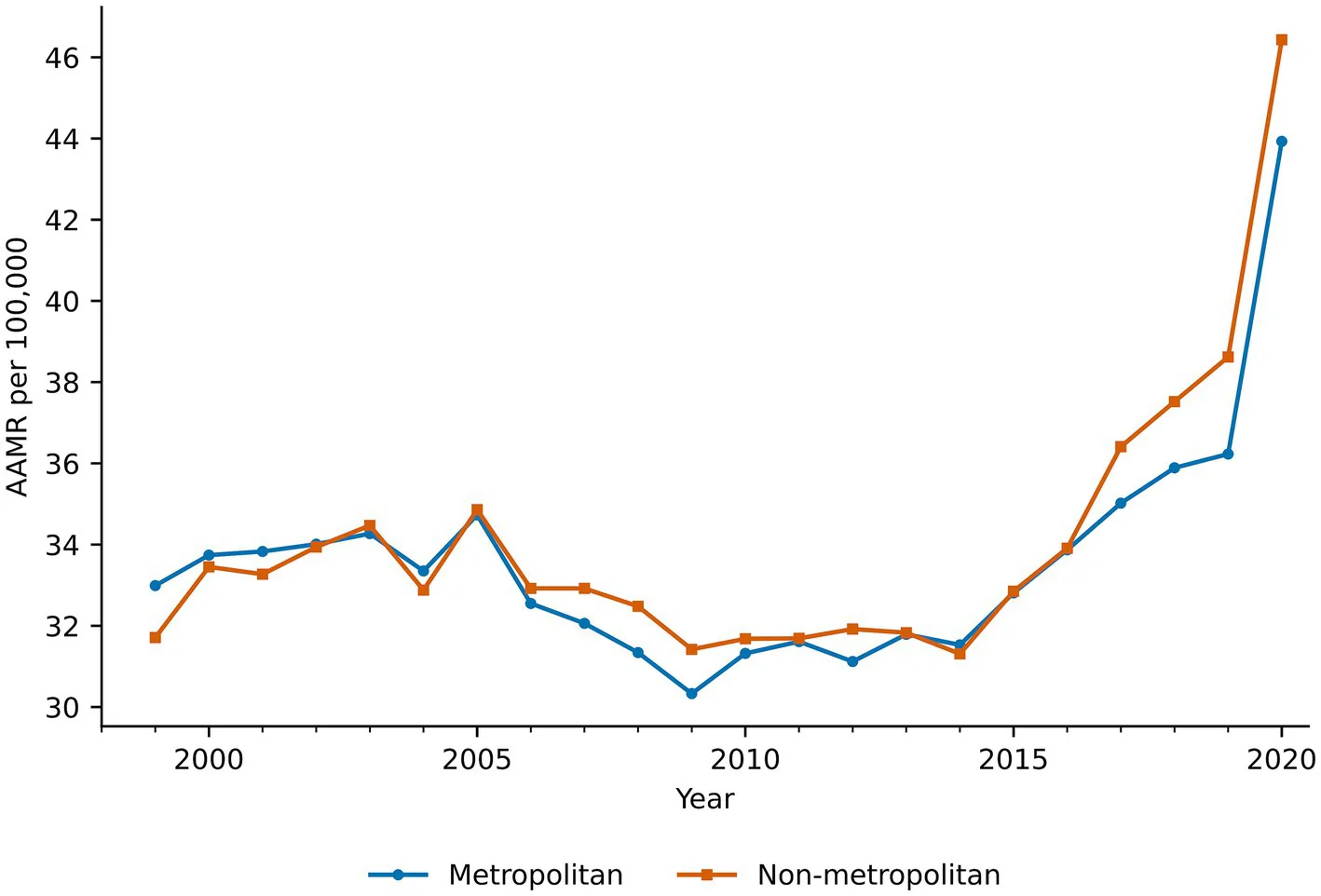
Age-adjusted Parkinson’s disease-related mortality by urbanization category, United States, 1999–2020. Observed data only; no projection is shown, because the NCHS urbanization series ends in 2020, its terminal observation coincides with the pandemic-year peak, and every candidate model extrapolated that peak to implausible values. Urbanization was defined using the 2013 NCHS urban–rural classification scheme for counties. AAMR, age-adjusted mortality rate; NCHS, National Center for Health Statistics.

Resolving urbanization to the full six-level NCHS classification shows a gradient concentrated at the rural end (Supplementary Table S10). Full-window AAPCs for 1999–2020 were 0.99% (95% CI 0.12 to 1.86) in large central metropolitan counties, 0.96% (0.40 to 1.53) in large fringe metropolitan, 0.80% (0.15 to 1.45) in medium metropolitan, 1.08% (0.61 to 1.54) in small metropolitan, 1.17% (0.59 to 1.75) in micropolitan, and 1.42% (0.88 to 1.96) in noncore counties. All six increased significantly, but the steepest rise was in the most rural stratum, and every stratum showed the same pattern of an early decline followed by a sharp late-period increase.

Urbanization projections are not reported under the reportability rule. The directional conclusion that the non-metropolitan excess widened through 2020 and warrants targeted surveillance rests on the observed data and is unaffected.

### 3.7 Census region and state

The Midwest had the highest AAMR in 23 of the 27 observed years, and the West had the highest in the remaining four (1999, 2001, 2003, and 2006) (Supplementary Table S11). The South showed the strongest full-window increase, from 29.55 to 42.72 (AAPC 1.06%; 95% CI 0.41 to 1.72; *p* = 0.001). Among the strata observed through 2025, the Northeast was the only region whose terminal joinpoint segment was rising, and this statement needs qualification. Its fitted model has a single inflection in 2011, so the terminal segment spans 14 years and contains the 2014–2021 surge; it is not a description of the recent direction of the series. Over the post-peak period, the Northeast rate fell from 40.56 in 2020 to 35.38 in 2025, a decline of 12.8%, the largest of the four regions. The Northeast is one of only two regions whose 2030 projection lies below its 2025 observation (34.08 against 35.38; the West is the other, at 38.96 against 39.71). The West, by contrast, had a flat full-window trend (AAPC 0.07%; −0.47 to 0.61; *p* = 0.800) and a significantly declining terminal segment (2021–2025 APC −2.65%; *p* = 0.026).

Projections for 2030 were 34.08 (95% PI 27.93–41.59) for the Northeast, 44.85 (35.86–56.09) for the Midwest, 46.74 (37.11–58.86) for the South, and 38.96 (32.63–46.51) for the West (Figure 5; Supplementary Table S11).

**Figure 5:**
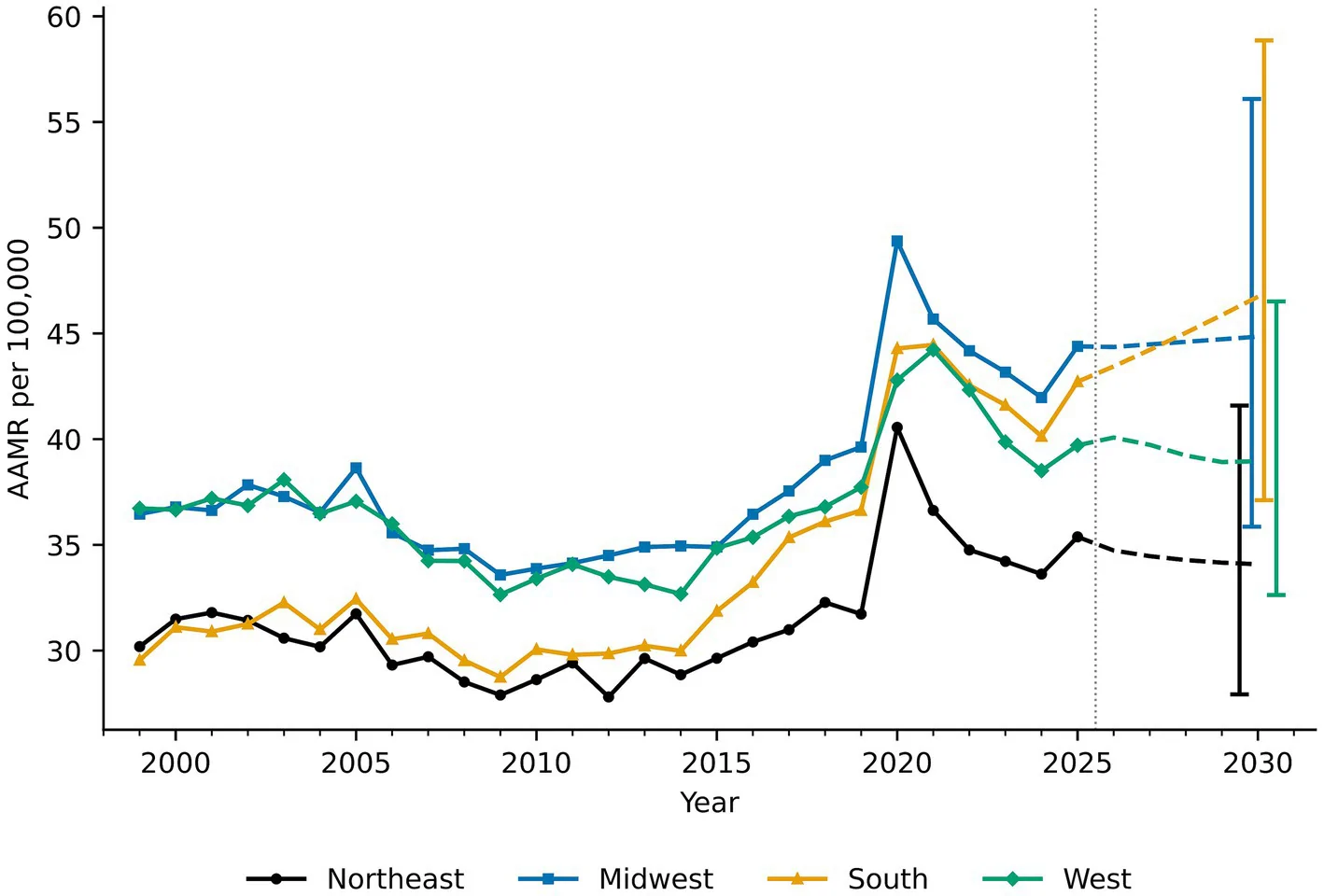
Age-adjusted Parkinson’s disease-related mortality by census region, United States, 1999–2030. Solid lines with markers represent observed AAMRs; dashed lines represent ensemble projections through 2030. Vertical bars at 2030 represent 95% prediction intervals, offset horizontally to avoid overlap. The dotted vertical line marks the boundary between observed and projected data. AAMR, age-adjusted mortality rate.

At the state level, Nebraska had the highest pooled AAMR in the 1999–2020 window (44.32). In the 2021–2025 window Nebraska (54.30; 95% CI 52.09–56.59) and Oklahoma (54.27; 52.67–55.91) were effectively tied, differing by 0.03 per 100,000 with almost completely overlapping confidence intervals; we report these as the two highest point estimates rather than ranking one above the other. The District of Columbia (24.62) and Hawaii (26.42) had the lowest point estimates in the respective windows (Supplementary Table S12). AAPC point estimates with 95% CIs for all strata are summarized in Figure 6.

**Figure 6:**
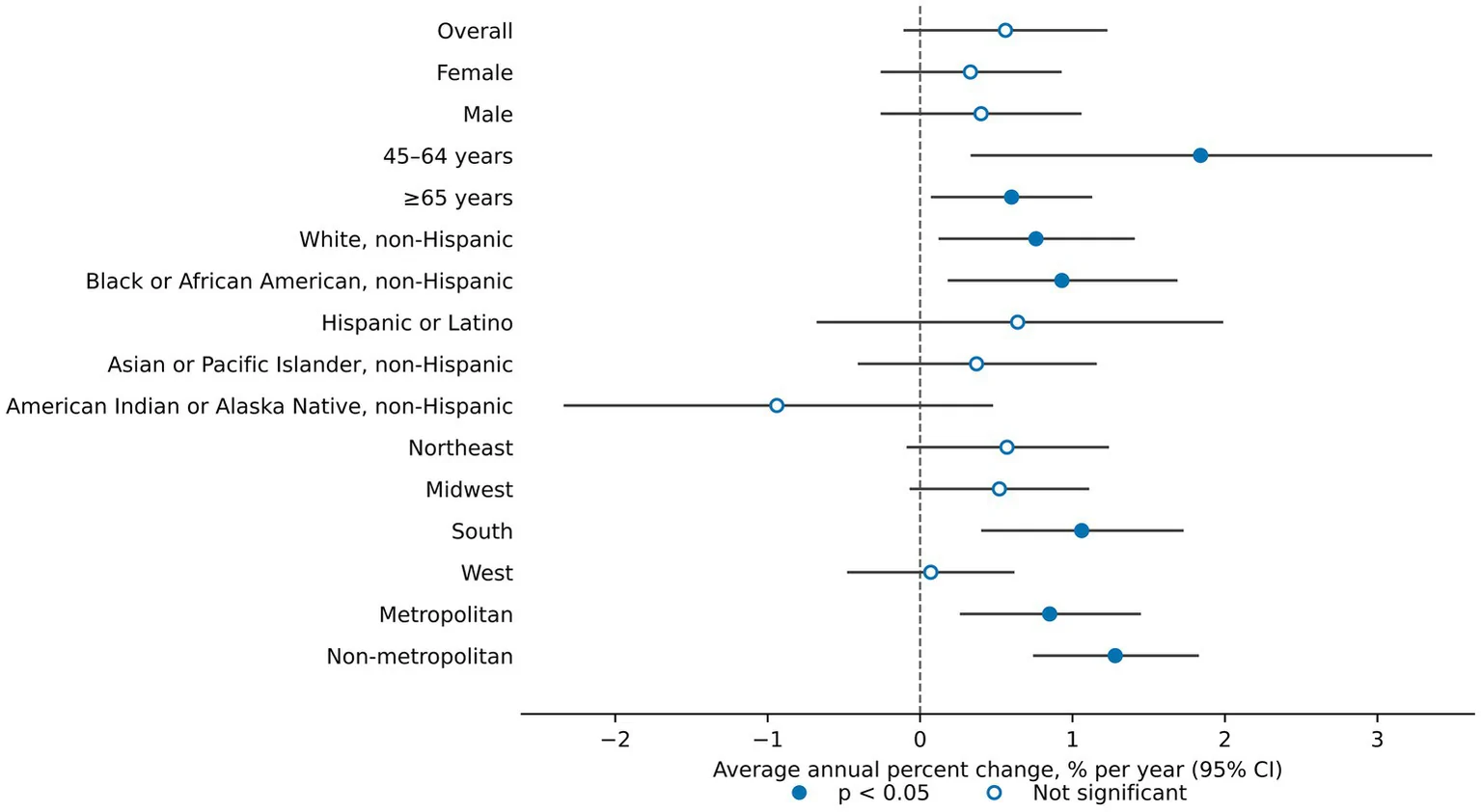
Average annual percent change (AAPC) in age-adjusted Parkinson’s disease-related mortality across demographic and geographic strata, United States, 1999–2025. Points are AAPC estimates from joinpoint regression; horizontal lines are 95% confidence intervals. Filled markers denote strata with *p* < 0.05; open markers denote non-significant trends. The dashed vertical line marks no change (AAPC = 0). Urbanization estimates cover 1999–2020; all other strata cover 1999–2025. Sixteen strata were tested without adjustment for multiplicity; therefore, these *p*-values are descriptive. AAPC, average annual percent change; CI, confidence interval.

### 3.8 Ensemble calibration

Calibration directly affects how projections should be read. Rolling-origin ensemble back-test error at the five-year horizon ranged from 8.6% (Hispanic or Latino) to 17.2% (adults aged 45–64), with the overall series at 13.4% (Supplementary Table S13). The error grew steadily with the horizon: for the overall series, 4.2% at 1 year, 13.4% at 5 years, and 24.0% at 10 years. All of these figures exclude 2020 and 2021 as back-test targets, as specified in Section 2.5; the calibration reported here is therefore calibration outside the pandemic years, and it would be worse if those years were scored.

Back-test bias under the primary specification was negative in 15 of 16 strata (range −13.7 to +0.3% at the five-year horizon), indicating that models trained on earlier origins tended to under-predict subsequent observed rates. This is the expected signature of a series containing an abrupt mid-window acceleration that no earlier training window can anticipate. The pre-pandemic-anchored specification is biased in the same direction, rather than the opposite. Its own rolling-origin bias at the five-year horizon is −11.4% for the overall series, and carried forward from the 1999–2019 window, it under-predicts every observed year from 2020 to 2025 (37.02, 37.25, 37.42, 37.57, 37.71, and 37.82 against observed values of 44.36, 43.13, 41.40, 40.16, 38.93, and 41.03; mean signed error −9.5%). Therefore, both specifications under-predict, and the choice between them cannot rest on a contrast of bias directions. We treat the full-data specification as primary on the simpler and sufficient ground that it uses all available observations, including the post-2021 decline, whereas the pre-pandemic anchor discards 6 years of data by design. The back-test bias for both specifications is reported in Supplementary Table S13.

In 14 of the 16 strata, the ARIMA member received the largest ensemble weight, reflecting its lower back-test error relative to both exponential smoothing members at the horizons tested; ARIMA weights ranged from 0.325 to 0.529 across the 16 strata. There are two exceptions in which the exponential smoothing member is the largest: the West (Holt 0.339, damped 0.327, ARIMA 0.333) and non-metropolitan counties (Holt 0.334, damped 0.341, ARIMA 0.325). In both cases, the three weights are near-equal, which is what inverse-error weighting returns when no member is clearly better in back-testing. Projections should be read as conditional planning estimates over a five-year horizon, not as precise predictions.

An independent ARIMA analysis, implemented separately in different software, provides a useful check (Supplementary Table S14). In 10 of the 14 projected strata that implementation selected a random walk without drift, for which the optimal forecast is the last observed value carried forward; its 2030 estimate in those strata is therefore the 2025 observation itself. Our ensemble, which additionally contains two trend components, lands a median of 1.7% away from the independent estimate across the 14 compared strata (1.6% across the ten random-walk strata alone; maximum 9.4% in the South), and the independent point estimate falls inside our 95% prediction interval in all 14 strata.

We draw a specific and limited inference from this. The agreement does not represent two independent trend estimates converging, because most of the independent estimates are not trend estimates at all. What it shows is that the trend components in our ensemble are not manufacturing drift: given a series with no detectable trend to extrapolate, our specification returns something very close to no change, which is the correct behavior and is consistent with the non-significant full-window AAPC. The limit of the check should also be stated: the two implementations converged on the same model class in most strata rather than reaching an agreement by different routes. In 8 of the 14 projected strata, the ARIMA member of our ensemble is likewise a random walk, (0,1,0) without trend, against 10 of 14 in the independent implementation. Therefore, a portion of the apparent concordance holds by construction rather than by independent corroboration.

### 3.9 COVID-19 co-listing

COVID-19 (U07.1) was recorded alongside PD on 17,657 death certificates between 2020 and 2025. Co-listing peaked in 2020, when 6,421 of 63,238 PD-mentioned deaths (10.15%) also listed COVID-19, and fell steadily thereafter: 7.17% in 2021, 6.18% in 2022, 3.03% in 2023, 1.84% in 2024, and 0.79% in 2025 (Supplementary Table S15). No PD-mentioned death listed U07.1 in 2019, as expected.

COVID co-listing accounted for 58% of the single-year rise in the overall AAMR between 2019 and 2020 (4.48 of 7.73 per 100,000). The residual non-COVID increase of 3.25 per 100,000, equivalent to a rise of 8.9% on the 2019 rate, occurred in the same year.

Subtracting co-listed deaths yields a PD-excluding-COVID series that is notably flat across the pandemic and post-pandemic period: 39.88 in 2020, 40.08 in 2021, 38.88 in 2022, 38.95 in 2023, 38.20 in 2024, and 40.71 in 2025. Against this, the observed decline in the all-PD series from 43.13 in 2021 to 38.93 in 2024 is substantially an unwinding of COVID co-listing: falling co-listed mortality accounts for 2.32 of the 4.20 per 100,000 decline (55%), while the non-COVID series fell only 4.7% over the same 3 years. The 2025 non-COVID value of 40.71 was the highest in that series.

### 3.10 Projected death counts

Applying the projected rates to Census Bureau population projections, PD-related deaths among adults aged ≥45 years are projected to rise from 62,162 observed in 2025 to 70,825 in 2030 (95% prediction interval 57,517 to 87,237), an increase of 13.9% (Supplementary Table S16).

Under the constant-rate scenario, in which the age-adjusted rate is held at its 2025 value and only the population ages, the projected 2030 count is 69,675, an increase of 12.1%. Population aging thus accounts for approximately 87% of the projected increase in deaths. Over the same interval the U.S. population aged ≥65 years is projected to grow by 12.4%, from 63.3 million to 71.2 million.

### 3.11 Case-definition sensitivity analyses

Restricting to deaths for which PD was the underlying cause yields a series that rises considerably faster than the any-mention series, not more slowly. The underlying-cause AAMR moved from 15.28 per 100,000 in 1999 to 28.45 in 2025 (+86.2%), against +25.3% for the any-mention series over the same window (Supplementary Table S17). The share of PD-mentioned deaths for which PD was recorded as the underlying cause rose from 46.7 to 69.2%, while the number of deaths in which PD was mentioned but was not the underlying cause was only 15.8% higher at the end of the window than at the start (16,532 in 1999; 19,142 in 2025), although it did not move monotonically (Section 4.2).

Restricting the joinpoint analysis to final data only (1999–2024, excluding the provisional year) left the conclusions unchanged (Supplementary Table S18). The overall AAPC was 0.51% (95% CI −0.21 to 1.23; *p* = 0.163) against 0.56% (−0.10 to 1.22; *p* = 0.099) for the full window, joinpoints remained at 2014 and 2021, and the terminal segment remained negative and non-significant, though steeper (APC −3.44%; −7.64 to 0.96; *p* = 0.116). The male stratum behaved similarly, with joinpoints unchanged in 2014 and 2021 and an AAPC of 0.34% (−0.34 to 1.02; *p* = 0.329) against 0.40% in the primary analysis. However, the female stratum did not show this trend. Its joinpoints moved from 2015 and 2020 in the primary analysis to 2014 and 2021 on final data only, and its AAPC fell from 0.33% (−0.25 to 0.92; *p* = 0.267) to 0.22% (−0.58 to 1.03; *p* = 0.586). The substantive reading is unchanged, the female full-window trend is non-significant in both fits, and the terminal segment is negative in both, but the inflection points in the female series are not stable to the exclusion of the provisional year, and the female estimates should be read with that in mind.

Adding secondary parkinsonism (G21) to the case definition changed the estimates negligibly. G21 contributed between 0.31 and 1.65% of annual deaths, and the mean absolute difference in AAMR across the 27 years was 0.40 per 100,000 (Supplementary Table S19). The increment is not constant across the window: it widens from +0.10 per 100,000 in 1999 to +0.68 in 2025, so adding G21 would slightly steepen the trend rather than shift the series by a fixed amount. The magnitude remains too small to alter any estimate or conclusion, and the G20-only definition is robust.

## 4 Discussion

In this 27-year national analysis, PD was recorded on 1,149,731 death certificates. The central finding is one of stability with a large interruption rather than sustained growth: the full-window trend in age-adjusted PD-related mortality was not statistically significant, and the record is dominated by a steep 2014–2021 surge between a modest earlier decline and a post-2021 decline that is now visible in 13 of the 14 strata observed through 2025. Near-term projections indicate a plateau, with the overall AAMR estimated at 41.73 per 100,000 in 2030 against 41.03 observed in 2025.

The direction and magnitude of a long-range projection depend heavily on specification. Ensemble weights permitted outside the unit interval, or a model fitted on an additive scale that admits negative rates, can generate a projected rise that reflects the extrapolation device rather than the data. We therefore constrain weights to be non-negative and to sum to one, fit on the log scale, and report no estimate beyond 2030, because 27 annual observations cannot validate a longer horizon.

Three features carry planning relevance. Mortality was concentrated among adults aged ≥65 years; it remained approximately 2.3-fold higher among men than women throughout, a ratio that was stable across 27 years; and a large share of deaths occurred in nursing homes, long-term care facilities, and private homes, situating PD mortality within long-term and community care systems rather than acute settings (5–7).

The persistence of the male excess is itself informative. A rate ratio that holds near 2.3 across 27 years, through a period in which the underlying rate fell, rose steeply, and fell again, is more consistent with stable underlying determinants than with changing ascertainment. Proposed mechanisms include biological susceptibility, sex hormone and neuroimmune differences, environmental and occupational exposures, traumatic brain injury risk, and sex-specific progression (8, 26). Both sexes showed the same three-phase temporal pattern, which suggests that the late-period surge reflected broad system- and population-level factors rather than sex-specific mechanisms; consistent with this, a meta-analysis of hospitalized patients found that the association between PD and adverse COVID-19 outcomes was modified by age but not by sex (27).

### 4.1 Relation to prior CDC WONDER analyses

Our findings sit alongside a small number of recent CDC WONDER analyses of PD mortality. Rong et al. (15) described underlying-cause mortality from 1999 to 2019, and Sokhal et al. (16) reported underlying-cause PD-related mortality through 2020; both were restricted to the underlying-cause database, ended before or at the post-2020 inflection, and did not forecast. Alam et al. (13) examined PD co-listed with mental-health disorders from 1999 to 2023 and reported a much lower rate reflecting that comorbidity-restricted definition. Hadidchi et al. (14) focused specifically on racial differences. Weresh et al. (12) also drew on the Multiple Cause-of-Death public use records, assessing PD as a contributing cause nationwide in adults aged ≥65 years from 1999 to 2022, and reported trends by sex, race, region, age, and place of death, together with machine-learning predictions. Their reported ≥65 series (88.9 in 1999 rising to 110.6 in 2022) tracks ours closely (88.62 in 1999; 111.17 in 2022), which supports convergent validity but also confirms substantial overlap. However, two of their results diverge from ours. Weresh et al. (12) reported that deaths at home decreased across their window, whereas we find the home share rising from 19.37 to 34.93%; the same direction of increase is reported by Kumar et al. (5) for 2003–2017, so we regard the rise as the better-supported result. They also identified the South as the region with the highest mortality, whereas we found the highest AAMR in the Midwest in 23 of the 27 years with the steepest increase in the South; the two statements are reconcilable if their ranking reflects the rate of change rather than the level of mortality. Our incremental contribution consists of three additional observation years, which is what makes the post-2021 decline visible; extension of the analytic population to adults aged ≥45 years; the urbanization stratum; explicit prediction intervals with reported back-test calibration; and a horizon chosen by empirical validation rather than convention.

Abid et al. (28) examined mortality involving co-recorded Parkinson’s disease and dementia among U.S. adults aged ≥45 years from 1999 through 2025, using the same Multiple Cause of Death database, the same any-mention ascertainment logic, and the same joinpoint approach applied in this study. The two analyses address different populations: Abid et al. (28) identified 337,721 deaths in which both G20 and a dementia code (F01, F03, G30, and G31) were recorded, whereas the present analysis identified all 1,149,731 deaths with any mention of G20, irrespective of co-recorded dementia. Their cohort is therefore a subset comprising 29.4% of ours, defined by a comorbidity restriction, and the two papers are complementary rather than overlapping in their findings. They reported an overall AAMR rising from 5.75 in 1999 to 11.15 in 2025 (AAPC 2.07; *p* = 0.002) with a sharp transient increase in 2020, followed by a significant decline through 2025, and the highest burden among males, non-Hispanic White individuals, the Midwest, and non-metropolitan areas, the same demographic and geographic gradient we observe. That an independent case definition applied to the same source over the same window produces the same terminal decline strengthens our confidence that the post-2021 decrease reported here is a feature of the data rather than of our modeling choices. They likewise caution that 2025 data are provisional.

Zhang et al. (29) analyzed mortality in type 2 diabetes with concomitant Parkinson’s disease (E11 with G20) from 1999 to 2023 and projected to 2030. That comorbidity-restricted population is again distinct from ours, but their choice of a 2030 rather than a mid-century horizon is consistent with the calibration limits described in the Methods.

### 4.2 Case definition and ascertainment

The most important threat to a rising any-mention rate is that the mean number of conditions recorded per U.S. death certificate has increased over the study window, so part of an apparent increase may reflect more complete certification rather than more PD-related death. However, a parallel underlying cause analysis did not support this. If the any-mention rise were driven by certificates listing more conditions over time, the growth would sit in the contributing-cause component and the underlying-cause series would be flatter than the any-mention series. The opposite is observed. The comparison is best drawn on the rate scale rather than on counts because counts confound the certification question with population growth and aging. Decomposing the any-mention age-adjusted rate into its two mutually exclusive components (Supplementary Table S17), the underlying-cause rate rose from 15.28 to 28.45 per 100,000 between 1999 and 2025 (+86.2%), while the contributing-only rate fell from 17.46 to 12.58 (−27.9%); the any-mention rate rose 25.3%, and the proportion of PD-mentioned deaths for which PD was the underlying cause rose from 46.7 to 69.2%. Neither component moved monotonically, and we state this explicitly because the endpoints alone can be misleading. The contributing-only rate declined for 15 years to a trough of 10.50 per 100,000 in 2014, recovered to 11.50 by 2019, rose to 16.26 in the pandemic year 2020, and fell back to 12.58 by 2025. On counts, the same component fell from 16,532 deaths in 1999 to 12,959 in 2014 and then rose 47.7% to 19,142 in 2025; a full-window count comparison shows a rise of only 15.8% only because 1999 was itself a local high, and the annual series does not support describing this component as static over any part of the window. What survives every framing is the relative movement of the two components: across the window, the underlying-cause rate rose by 86%, while the contributing-only rate ended below where it began.

The certification shift over this period has therefore been in how PD is *ranked* among recorded causes, not in whether additional conditions are listed. That shift inflates an underlying-cause series substantially, and the any-mention definition we use is invariant to it: a death is counted whether PD is recorded as the underlying or a contributing cause. On this evidence the any-mention definition is the more conservative and more stable of the two, and the specific ascertainment mechanism the concern rests on is not supported by these data.

The scope of this finding is limited. This rules out the hypothesis that a lengthening cause-of-death list inflated the numerator, but not the broader possibility that PD is recorded on death certificates more often now than in 1999 as clinical recognition has improved, which would affect both definitions equally and which no death-certificate analysis can resolve. This caveat is discussed in the Limitations section. The analysis also renders our estimates directly comparable with those of Rong et al. (15) and Sokhal et al. (16), who used the underlying cause database.

This concern also bears on our interpretation of racial and ethnic patterns and complicates a simple reading. Lower recorded mortality in Black populations should not be interpreted as a lower underlying disease burden because diagnosis and death-certificate coding are shaped by access to neurologic care, diagnostic delay, referral patterns, treatment availability, competing mortality, and clinician recognition of Parkinsonism across groups (9, 10, 30). But the same mechanism means that the comparatively large proportional rise among Black or African American decedents cannot be attributed to a worsening epidemiological burden without also considering improving diagnostic recognition and certification. These two explanations cannot be distinguished using death certificate data alone. The pattern among Asian or Pacific Islander individuals may also conceal substantial heterogeneity across Asian American, Native Hawaiian, and Pacific Islander subgroups that broad administrative categories cannot resolve (10, 30)—a limitation compounded, in our data, by the mid-window change in how CDC WONDER codes these groups.

### 4.3 The 2014–2021 surge and the post-2021 decline

The 2014–2021 rise is the dominant feature of the record, and its interpretation determines what the near-term projections mean. Quantifying the COVID-19 contribution is therefore essential, and the measurement is informative in three respects.

First, COVID-19 co-listing accounts for 58% of the 2019–2020 jump, not all of it. A residual non-COVID increase of 8.9% occurred in the same single year, which is large by the standards of this study. Part of it may reflect uncertified COVID-19: hospitalized patients with PD had roughly 2.6-fold higher odds of severe disease and death than those without in a meta-analysis (27), so infection may have precipitated deaths on which U07.1 was never recorded. Indirect pandemic effects are also plausible, including disrupted access to neurologic and rehabilitative care, staffing shortages in long-term care, deferred management of dysphagia and falls, and reduced caregiver support among a population in which a large share resides in nursing homes, although these cannot be quantified in death certificate data. Part of the rise also predates 2020 entirely, since the joinpoint segment begins in 2014. A second and independent trace of the same mechanism appeared in the case definition data. The proportion of PD-mentioned deaths for which PD was the underlying cause had risen in every year to 68.5% in 2019; in 2020, it fell to 63.2%, the only interruption in the whole series, and then recovered to 65.2, 65.9, 67.9, and 68.5 to 69.2% in 2025 (Supplementary Table S17). A displacing cause that takes the underlying-cause position on certificates that still mention PD is precisely what produces that signature, and its timing coincides exactly with the co-listing peak in Supplementary Table S15. The two supplementary series were derived from separate extractions and corroborated each other.

Second, and more consequentially for how the terminal decline should be read, most of that decline is COVID unwinding rather than improvement in PD-related mortality. Falling co-listing accounts for 55% of the 4.20 per 100,000 decrease between 2021 and 2024, and the PD-excluding-COVID series is close to flat across the entire pandemic and post-pandemic period (39.88 in 2020, 40.08 in 2021, 38.20 in 2024, 40.71 in 2025). The apparent recovery in the headline series is therefore substantially the withdrawal of an additive COVID component, superimposed on an underlying rate that has changed little since 2020.

Third, that mechanism is now nearly exhausted. COVID co-listing has fallen to 0.79% of PD-mentioned deaths in 2025, so there is very little COVID contribution left to unwind. Any further movement in the headline series will therefore largely reflect the underlying PD-related mortality rather than the pandemic component. This independently supports the plateau our projections indicate, and it argues against extrapolating the 2021–2024 decline forward — which is precisely what the pre-pandemic-anchored specification, and any model fitted naively to the terminal segment, would do.

The subsequent decline is now observable across almost the entire stratification. Whether this represents recovery from a transient disruption, stabilization of reporting, or genuine change cannot be determined with these data, and post-2025 surveillance will be needed to distinguish them (12). The terminal year requires particular comments. Data through 2024 are final, whereas 2025 are provisional, and 2025 shows both more deaths (62,162 vs. 59,018) and a higher AAMR (41.03 vs. 38.93) than 2024 after four consecutive years of decline. Because provisional files are incomplete relative to their final release, the direction of the expected bias is downward, so a provisional year that already exceeds the preceding final year is unlikely to be an artifact of undercounting. We were able to measure this rather than assert it because the sensitivity analyses required a second extraction 6 weeks after the first (Methods 2.1). Between June 26 and August 7, 2026, the recorded 2025 counts rose in all four non-Hispanic race categories: non-Hispanic White from 52,634 to 52,716, non-Hispanic Black or African American from 3,224 to 3,233, non-Hispanic American Indian or Alaska Native from 206 to 207, and non-Hispanic Asian or Pacific Islander from 2,106 to 2,116, a total of 102 additional deaths (+0.18%), with no category falling. Over the same 6 weeks, the 2024 counts, which are final, were identical to the digit in all four categories. The provisional year is still accruing records, while the final year is fixed; therefore, the 2025 rate reported here is more likely to be revised upward than downward. On that reading the terminal decline may have halted in 2025 rather than continued, which is a further reason to describe the current direction as uncertain rather than declining. The co-listing analysis reinforces this reading: the 2025 increase is not a COVID artifact, since co-listed deaths fell to their lowest level that year, and the PD-excluding-COVID rate rose to 40.71, the highest value in that series. We nevertheless report the overall AAPC and the 2021–2025 segment with the provisional year excluded in Supplementary Table S18, because provisional and final files can also differ in the completeness of cause-of-death coding rather than in counts alone, and readers should be able to see the trend estimated on final data only.

Rates were highest, and rose most in absolute terms, among adults aged ≥65 years. The 45–64 stratum warrants separate comment: it showed the steepest full-window proportional increase of any age group (AAPC 1.84%; *p* = 0.016) from a very low absolute rate, and it was also the first stratum to turn significantly downward after 2021 (APC −2.71%; *p* = 0.045). Continued surveillance of midlife PD is warranted, particularly given the evidence of shifting early onset disease burden internationally (31).

### 4.4 Standardized rates, counts, and capacity planning

AAMRs are standardized to the 2000 U.S. population, so demographic aging is removed from them by construction and cannot explain their movement. Capacity planning, however, depends on the number of deaths and not on the standardized rate. We therefore report projected death counts alongside projected rates (Supplementary Table S16). Against 62,162 PD-related deaths observed in 2025, the primary specification projects 70,825 deaths in 2030 (95% prediction interval 57,517 to 87,237), an increase of 13.9%. This interval is wide, and its lower bound lies below the 62,162 deaths observed in 2025; the rate-based projection alone cannot exclude a five-year period with no growth in the number of deaths at all.

The decomposition is the substantive point. Holding the age-standardized rate exactly at its 2025 value and allowing only the population to age, the projected 2030 count is 69,675, an increase of 12.1%. Population aging therefore accounts for approximately 87% of the projected rise in the number of deaths, and the projected change in the standardized rate for the remaining 13%. Because this scenario holds the rate fixed, it carries only the uncertainty in the Census Bureau’s population projections and none of the forecast uncertainty in the rate; it is therefore the firmer of the two figures for capacity planning, and we treat it as the planning anchor, with the rate-based projection indicating that the epidemiological component adds little to it. Stated plainly, the great majority of the additional PD-related mortality burden that U. S. health systems should expect over the next 5 years arises from the increased number of older adults, not from any deterioration in age-specific mortality.

This reframes what our flat rate means for planning. A plateau in the AAMR is not reassuring from a capacity standpoint, because the U.S. population aged ≥65 years is projected to grow by 12.4% between 2025 and 2030. The planning implications of this paper rest on that arithmetic rather than on a rising rate, and they are if anything strengthened by it: the projected increase follows from demographic change, which is more predictable than a mortality trend and therefore a firmer basis for provisioning long-term care, home health, and palliative-care capacity. The two rate specifications bracket a modest planning range: 41.73 per 100,000 under the full-data model and 38.27 under the pre-pandemic anchor, a difference of approximately 9%. That spread is narrower than either specification’s own prediction interval, so the choice between them is a second-order consideration. The demographic component is both larger and more predictable, which is why we anchor planning on it.

### 4.5 Geographic and end-of-life patterns

Both metropolitan and non-metropolitan areas showed rising mortality through 2020, and the non-metropolitan excess widened in the late period, consistent with older age structure, reduced access to movement-disorder specialists, sparser rehabilitation resources, transportation barriers, delayed diagnosis, and constrained long-term care (11, 12). Willis et al. (32) quantified the specialist-access component directly in Medicare data: only 58% of incident PD cases received neurologist care, with women (OR 0.78, 95% CI 0.76–0.80) and non-White beneficiaries (OR 0.83, 0.79–0.87) less likely to receive it, and neurologist care was associated with lower skilled-nursing-facility placement (OR 0.79) and lower mortality (HR 0.78). This argues for expanded tele-neurology, home-health support, medication management, rehabilitation access, caregiver training, and stronger rural referral pathways. Regionally, the Midwest carried the highest burden in all but 4 years; state-level differences likely reflect variation in population aging, exposures, rurality, healthcare access, diagnostic recognition, and death-certification practice, and should be read as signals for place-based planning rather than evidence of geographic causation (11, 14). The projected 2030 rates preserve this spread and shift its ordering: the South is estimated at 46.74 per 100,000 and the Midwest at 44.85, against 38.96 in the West and 34.08 in the Northeast, a South-to-Northeast difference of approximately 37%. Because regions were modeled independently, these are stratum-specific directional estimates whose differences carry no valid joint uncertainty, and they indicate where the burden is likely to remain concentrated rather than constituting a formal test of divergence.

The place-of-death distribution is an important planning signal, and it changed substantially across the study window. Pooled across 27 years the distribution obscures that change, so we report it by period.

The hospice share of PD-related deaths rose from 0.88% in 1999–2007 to 6.21% in 2008–2016 and 8.25% in 2017–2025, a more than ninefold increase. The pooled figure understates current hospice use by roughly 2.5 percentage points and is an artifact of averaging across a period in which U.S. hospice provision expanded substantially. What the data show is rapid growth in hospice use among people dying with PD, not underuse.

The more consequential shift is in where these deaths now occur. Institutional deaths have markedly decreased. Nursing home and long-term care from 49.24 to 35.48%, inpatient facilities from 22.68 to 12.93% while home deaths have risen from 19.37 to 34.93%. This extends an established pattern rather than establishing it. Kumar et al. (5) documented the same redistribution in CDC WONDER underlying-cause data for 2003–2017, with nursing-home deaths falling from 52.6 to 42.0%, home deaths rising from 21.1 to 32.4%, and hospice deaths from 0.3 to 8.6%, and Zhang et al. (29) observed a comparable institutional-to-home shift through 2023. What the present series adds is continuation to 2025 under an any-mention definition, showing that the shift persisted through the pandemic and that home and long-term care have now converged. In the current period, home and long-term care are of almost equal magnitude, whereas at the start of the window institutional settings outnumbered home deaths by more than two to one. This is consistent with the advanced-stage course of PD, in which progressive immobility, dysphagia, aspiration risk, falls, dementia, autonomic dysfunction, and caregiver dependence drive intensive support needs (5–7), but it relocates those needs. The planning implication is not primarily about institutional bed capacity; it is about home-based care — informal caregiver burden, home health, community palliative services, and the infrastructure needed to support dying at home safely (5, 6, 33–36). The reorientation is not confined to the United States; Wilson et al. (36) found that care-home deaths in England and Wales fell significantly after March 2020, with a corresponding rise in home deaths, indicating a common post-pandemic pattern in Parkinson’s disease rather than a U.S.-specific artifact.

A randomized trial of integrated outpatient palliative care in Parkinson’s disease and related disorders reported a better quality of life, lower non-motor symptom burden, and higher completion of advance directives (33). Complementary work has developed tools for the timely identification of palliative care needs (34) and has set out the ethical considerations in advance care planning (35). However, the evidence base for this approach remains limited.

### 4.6 Implications

First, a plateau in the standardized rate combined with a growing older population still implies growing absolute demand, supporting the expansion of movement disorder and geriatric neurology capacity, rehabilitation, fall prevention, dysphagia management, cognitive and psychiatric support, caregiver services, and palliative care access (1, 5–7, 33–35). Second, the migration of these deaths out of institutions and into the home indicates a need for care models that integrate neurology, primary care, rehabilitation, long-term care, home health, hospice, and palliative care rather than relying on outpatient neurology alone, with the balance of investment shifting toward home and community-based provision. Third, geographic and urbanization patterns argue for place-based planning, including tele-neurology and structured rural referral networks. The urbanization component of that case rests on observed data through 2020 alone, because no urbanization projection met the reportability criteria, and the widening non-metropolitan excess should be re-examined as post-2020 urbanization data become available. Fourth, racial and ethnic differences should prompt renewed efforts toward diagnostic equity, expanded research representation and disaggregated reporting (9, 10, 30, 32). These recommendations differ in terms of the lead time they require. Capacity that can be reallocated within a few years—home health, caregiver support, community palliative services — is well matched to a five-year projection horizon, whereas subspecialty workforce expansion operates on training cycles longer than the horizon these data can validate and should be planned against demographic projections, which extend further and are more stable, rather than against our mortality forecast.

Earlier diagnosis and improved specialist access are desirable on their own terms, but under an any-mention case definition better recognition of PD would plausibly *increase* recorded PD-related mortality rather than reduce it. Recorded mortality is therefore a poor metric against which to judge the success of such interventions, and the same mechanism we invoke in the Limitations section applies here.

Future work should determine whether the observed changes reflect incidence, recognition, survival, certification practice, pandemic disruption, or frailty; linking mortality records with Medicare, nursing home, hospitalization, prescription, and neurologist-access data would strengthen causal interpretation (5–7, 34, 35).

## 5 Limitations

This analysis relied on death-certificate coding; estimates may be influenced by diagnostic accuracy, certification practice, coding conventions, and changing awareness over time (12, 14, 15). PD may be under-recorded when deaths are attributed to immediate complications (pneumonia, aspiration, falls, dementia, sepsis, cardiovascular events), while broader awareness may increase its documentation as a contributing cause. Because deaths were included whenever G20 appeared anywhere on the certificate, these estimates capture PD-related mortality rather than deaths for which PD was the sole underlying cause; the parallel underlying-cause analysis in Section 4.2 addresses how much this matters.

CDC WONDER changed from bridged-race to single-race coding during the study window, and the single-race files report Asian separately from Native Hawaiian or Other Pacific Islander. The harmonization we applied is stated in Section 2.2, but bridging reassigns multiple race descendants to one of four race groups, whereas single-race coding sequesters them. Therefore, both counts and their population denominators are systematically smaller after 2020 for the groups with the highest multiple-race reporting, and rates are not directly comparable across the break; the American Indian or Alaska Native series, which has the smallest annual cells of any stratum, is the most exposed to this problem. Race and ethnicity categories may additionally conceal heterogeneity by ancestry, nativity, socioeconomic status, and healthcare access (9, 10, 30); the combined Asian or Pacific Islander category is a concrete instance, dominated by its Asian component. Hispanic or Latino estimates derive from a direct query on the Hispanic Origin field. Records with Hispanic origin not stated (1,910 deaths, 0.166% of the analytic population) and, in 2021–2025, records reporting more than one race (859 deaths, a category with no bridged race counterpart) were not redistributed. As set out in Section 2.2, the 1,400 unstated-origin records from 1999 to 2020 are contained within the four non-Hispanic race series rather than excluded from them, and only the 510 from 2021 to 2025 fall outside all five categories. Therefore, all race and ethnicity estimates are conditional on the origin being recorded. The five categories exceeded the analytic total by 149 deaths (0.013%). This excess cannot be an artifact of the exclusions, which could only produce a deficit; it arises because the Hispanic-origin series was obtained by a direct query performed after the race extraction (Methods 2.1), so the two are of different data vintages. It is confined to the single-race years 2021–2025, the categories reconcile exactly with the annual totals in every year from 1999 to 2020, and the residual affects only the cumulative counts because every rate and trend was estimated within each stratum.

Data for 1999–2024 are final and data for 2025 are provisional, which could affect the terminal-year rate and the 2021–2025 segment; a sensitivity analysis excluding 2025 is reported in Supplementary Table S18. State-level data were analyzed across separate windows because of post-2020 reliability rules. Sixteen strata and 51 state estimates were tested without multiplicity adjustment.

Forecasts are conditional on historical patterns and do not incorporate future changes in incidence, treatment, care delivery, coding, or population composition. Because subgroup series were forecast independently, stratum projections are directional rather than an additive decomposition, and projected between-group differences carry no valid joint uncertainty. Back-test error at the five-year horizon exceeded 10% for most strata, so prediction intervals should be taken seriously rather than treated as a formality. Urbanization was not projected at all, for the reasons given in Section 3.6. The projected death counts inherit all of the uncertainty in the projected rates and add to it the assumption that the age composition within each broad age band is stable, so that the age-standardized rate ratio transfers to the band’s crude rate; they also depend on the accuracy of the Census Bureau’s population projections, which carry their own uncertainty that we have not propagated.

## 6 Conclusion

Age-adjusted PD-related mortality in the United States did not change significantly between 1999 and 2025 taken as a whole. The record is dominated by a pronounced 2014–2021 surge on an otherwise flat-to-declining baseline, followed by a decline that is now visible in almost every stratum, and the current direction remains uncertain. Near-term projections indicate a plateau near current levels rather than continued growth, and we do not project beyond 2030 because these data cannot validate a longer horizon.

The burden was not evenly distributed: mortality remained approximately 2.3-fold higher among men, was concentrated among adults aged ≥65 years, was highest among non-Hispanic White decedents, and showed a widening non-metropolitan excess through 2020. Because standardized rates remove population aging by construction, a flat rate combined with a growing older population still implies rising absolute numbers of PD-related deaths: we project 70,825 deaths in 2030 against 62,162 in 2025, with approximately 87% of that increase attributable to demographic change alone. Where these deaths occur has shifted substantially: institutional deaths have fallen while home and hospice deaths have risen, so that home and long-term care are now of almost equal magnitude. This relocates rather than reduces the need for support and argues for stronger access to movement disorder specialists, rehabilitation, tele-neurology for underserved areas, home health and caregiver support, and timely palliative care integration.

## Data Availability

All data analyzed in this study are publicly available and do not require special permission. Mortality data were obtained from the CDC WONDER Multiple Cause of Death Database (https://wonder.cdc.gov/mcd.html).
