# Supplementary material for: Parkinson’s disease mortality in the United States, 1999–2025: a national multiple-cause-of-death analysis with projections to 2030
Source: Front Neurol. 2026 Oct 1;17:1934146. doi: 10.3389/fneur.2026.1934146 (PMC13668596; doi:10.3389/fneur.2026.1934146)
Supplement: Supplementary file 1 [file Table_1.DOCX]

**Supplementary Material**

**Parkinson’s disease mortality in the United States, 1999–2025: a national multiple-cause-of-death analysis with projections to 2030**

**Appendix A. STROBE Checklist**

STROBE Statement; checklist of items that should be included in reports of observational studies, completed for the present serial cross-sectional analysis of national death-certificate data (von Elm E, et al. PLoS Med. 2007;4:e296; reference 19 of the main manuscript).

| **Item No.** | **STROBE recommendation** | **Reported in manuscript (section)** |
| --- | --- | --- |
| **Title and Abstract** | | |
| **1** | (a) Indicate the study’s design with a commonly used term in the title or the abstract. (b) Provide in the abstract an informative and balanced summary of what was done and what was found. | Title; Abstract |
| **Introduction** | | |
| **2** | Background/rationale: Explain the scientific background and rationale for the investigation being reported. | Introduction (paragraphs 1-4) |
| **3** | Objectives: State specific objectives, including any prespecified hypotheses. | Introduction (final paragraph) |
| **Methods** | | |
| **4** | Study design: Present key elements of study design early in the paper. | Methods - Study design and data source |
| **5** | Setting: Describe the setting, locations, and relevant dates, including periods of recruitment, exposure, follow-up, and data collection. | Methods - Study design and data source (CDC WONDER Multiple Cause of Death; United States; 1999-2025) |
| **6** | Participants: Give the eligibility criteria and the sources and methods of selection of participants. | Methods - Study design and data source; Population and stratification |
| **7** | Variables: Clearly define all outcomes, exposures, predictors, potential confounders, and effect modifiers. | Methods - Population and stratification; Outcome measures |
| **8** | Data sources/measurement: For each variable of interest, give sources of data and details of methods of assessment (measurement). | Methods - Study design and data source; Outcome measures |
| **9** | Bias: Describe any efforts to address potential sources of bias. | Methods - Outcome measures; Forecasting analysis; Limitations |
| **10** | Study size: Explain how the study size was arrived at. | Methods - Population and stratification (all recorded U.S. PD-related deaths, 1999-2025); Results |
| **11** | Quantitative variables: Explain how quantitative variables were handled in the analyses. | Methods - Population and stratification; Outcome measures; Trend analysis |
| **12** | Statistical methods: (a) Describe all statistical methods, including those used to control for confounding. (b) Describe methods used to examine subgroups and interactions. (c) Explain how missing/suppressed data were addressed. (d) Describe sensitivity analyses. | Methods - Trend analysis; Forecasting analysis; Software and visualization |
| **Results** | | |
| **13** | Participants: (a) Report numbers of individuals at each stage of the study. (b) Give reasons for non-participation. (c) Consider use of a flow diagram. | Results - Overall mortality burden and place of death (n = 1,149,731). (b-c) Not applicable: complete national death-certificate census. |
| **14** | Descriptive data: (a) Give characteristics of study participants and information on exposures and potential confounders. (b) Indicate number with missing data. (c) Summarise follow-up period. | Results - Overall mortality burden and place of death; Table 1; Supplementary Tables S2–S5. (c) Not applicable: cross-sectional mortality surveillance. |
| **15** | Outcome data: Report numbers of outcome events or summary measures. | Results; Table 1; Supplementary Tables S2, S11–S14 |
| **16** | Main results: (a) Give unadjusted and, where applicable, adjusted estimates and their precision (e.g., 95% CI). (b) Report category boundaries when continuous variables were categorized. (c) Translate relative risk into absolute risk for a meaningful time period where relevant. | Results - Table 1 (AAMRs and AAPCs with 95% CIs); Figures 1-6 |
| **17** | Other analyses: Report other analyses done - e.g., analyses of subgroups and interactions, and sensitivity analyses. | Results - demographic and geographic strata; Ensemble model calibration; Supplementary Tables S7, S9, S4, S8, S16, S19 |
| **Discussion** | | |
| **18** | Key results: Summarise key results with reference to study objectives. | Discussion (first paragraph) |
| **19** | Limitations: Discuss limitations of the study, taking into account sources of potential bias or imprecision. | Limitations |
| **20** | Interpretation: Give a cautious overall interpretation of results considering objectives, limitations, multiplicity of analyses, results from similar studies, and other relevant evidence. | Discussion; Forecast interpretation and projection uncertainty |
| **21** | Generalisability: Discuss the generalisability (external validity) of the study results. | Discussion; Limitations |
| **Other Information** | | |
| **22** | Funding: Give the source of funding and the role of the funders for the present study and, if applicable, for the original study on which the present article is based. | Declarations - Funding statement |

**List of Supplementary Tables**

Supplementary Table S1. Place of death, pooled and by period, 1999–2025.

Supplementary Table S2. Trends in age-adjusted mortality rates and deaths, overall and by sex, 1999–2025.

Supplementary Table S3. Annual percent change and average annual percent change by stratum, 1999–2025.

Supplementary Table S4. Projected age-adjusted mortality rates with 95% prediction intervals, 2026–2030.

Supplementary Table S5. Trends in age-adjusted mortality rates and deaths by age group, 1999–2025.

Supplementary Table S6. Trends in age-adjusted mortality rates and deaths by race and ethnicity, 1999–2025.

Supplementary Table S7. Hispanic-origin distribution from direct query, 1999–2025.

Supplementary Table S8. Single-race detail underlying the combined Asian or Pacific Islander category, 2018–2025.

Supplementary Table S9. Trends in age-adjusted mortality rates and deaths by urbanization, 1999–2020.

Supplementary Table S10. Six-level NCHS urbanization gradient, 1999–2020.

Supplementary Table S11. Trends in age-adjusted mortality rates and deaths by census region, 1999–2025.

Supplementary Table S12. Age-adjusted mortality rates and deaths by state, 1999–2020 and 2021–2025.

Supplementary Table S13. Ensemble composition, selected ARIMA order, and rolling-origin back-test performance.

Supplementary Table S14. Independent replication: 2030 projections from a separately implemented ARIMA analysis.

Supplementary Table S15. COVID-19 (U07.1) co-listing among PD-mentioned deaths, 2019–2025.

Supplementary Table S16. Projected death counts, 2026–2030, with a constant-rate scenario.

Supplementary Table S17. Ascertainment sensitivity: PD as underlying cause versus any mention, 1999–2025.

Supplementary Table S18. Joinpoint trend estimates restricted to final data, 1999–2024.

Supplementary Table S19. Case-definition sensitivity: G20 alone versus G20 with G21.

**Supplementary Table S1.** Place of death among Parkinson’s disease-related deaths, U.S. adults aged ≥45 years, pooled and by period.

| Place of death | 1999–2007, % | 2008–2016, % | 2017–2025, % | Pooled 1999–2025, % | Pooled deaths |
| --- | --- | --- | --- | --- | --- |
| Nursing home/long term care | 49.24 | 42.31 | 35.48 | 41.11 | 472,688 |
| Decedent’s home | 19.37 | 27.28 | 34.93 | 28.58 | 328,650 |
| Medical Facility - Inpatient | 22.68 | 15.53 | 12.93 | 16.26 | 186,940 |
| Hospice facility | 0.88 | 6.21 | 8.25 | 5.71 | 65,677 |
| Other | 4.21 | 5.76 | 6.40 | 5.63 | 64,759 |
| Medical Facility - Outpatient or ER | 2.87 | 2.42 | 1.92 | 2.32 | 26,679 |
| Medical Facility - Dead on Arrival | 0.46 | 0.15 | 0.08 | 0.20 | 2,305 |
| Place of death unknown | 0.16 | 0.34 | 0.00 | 0.14 | 1,639 |
| Medical Facility - Status unknown | 0.13 | 0.00 | 0.00 | 0.03 | 394 |

Percentages are of all PD-related deaths within the stated period. Deaths were identified by ICD-10 G20 recorded anywhere on the death certificate, adults aged ≥45 years; 1999–2020 from the Multiple Cause of Death 1999–2020 file and 2021–2025 from the Multiple Cause of Death 2018–2025 (Single Race) file; both extracted on 26 June 2026 (Methods 2.1 lists every extraction and its date). The two “unknown” rows reflect how CDC WONDER codes and releases the place-of-death field rather than a change in reporting behaviour. “Medical Facility – Status unknown” is populated only in 1999–2002 (85, 97, 101 and 111 deaths) and is empty in every year from 2003 onward, when it ceased to be a reportable category. “Place of death unknown” falls to 10 deaths in 2017, 10 in 2018 and none reported in 2019–2025, so the 0.00% shown for 2017–2025 corresponds to 20 deaths in total across nine years; because CDC WONDER suppresses cells containing fewer than ten deaths, “none reported” in the later years is consistent with anything between zero and nine deaths per year and should be read as a suppression floor rather than a demonstrated zero.

**Supplementary Table S2: Trends in Age-Adjusted Mortality Rates and Deaths from Parkinson’s Disease among United States Adults, stratified by Overall and Sex, 1999–2025.**

| **Year** | **Overall** | **Overall** | **Female** | **Female** | **Male** | **Male** |
| --- | --- | --- | --- | --- | --- | --- |
| **Year** | **Deaths** | **AAMR (95% CI)** | **Deaths** | **AAMR (95% CI)** | **Deaths** | **AAMR (95% CI)** |
| 1999 | 31,010 | 32.74 (32.38–33.11) | 13,633 | 22.48 (22.10–22.86) | 17,377 | 50.85 (50.08–51.62) |
| 2000 | 32,293 | 33.66 (33.29–34.03) | 14,215 | 23.24 (22.86–23.63) | 18,078 | 52.30 (51.53–53.08) |
| 2001 | 32,871 | 33.73 (33.36–34.09) | 14,320 | 23.12 (22.74–23.50) | 18,551 | 52.33 (51.57–53.10) |
| 2002 | 33,527 | 34.01 (33.64–34.37) | 14,664 | 23.48 (23.10–23.86) | 18,863 | 52.34 (51.58–53.10) |
| 2003 | 34,353 | 34.29 (33.93–34.66) | 14,976 | 23.67 (23.29–24.05) | 19,377 | 52.45 (51.70–53.20) |
| 2004 | 33,716 | 33.25 (32.89–33.60) | 14,498 | 22.75 (22.38–23.12) | 19,218 | 51.04 (50.31–51.77) |
| 2005 | 35,861 | 34.75 (34.39–35.10) | 15,477 | 23.88 (23.50–24.26) | 20,384 | 52.89 (52.16–53.63) |
| 2006 | 34,265 | 32.59 (32.24–32.94) | 14,568 | 22.13 (21.77–22.49) | 19,697 | 49.82 (49.12–50.52) |
| 2007 | 34,443 | 32.21 (31.87–32.55) | 14,647 | 22.03 (21.67–22.39) | 19,796 | 48.86 (48.17–49.54) |
| 2008 | 34,312 | 31.53 (31.19–31.86) | 14,391 | 21.36 (21.01–21.72) | 19,921 | 48.03 (47.36–48.70) |
| 2009 | 33,766 | 30.52 (30.20–30.85) | 13,936 | 20.40 (20.06–20.75) | 19,830 | 46.73 (46.07–47.38) |
| 2010 | 35,175 | 31.38 (31.05–31.71) | 14,615 | 21.08 (20.74–21.43) | 20,560 | 47.50 (46.84–48.15) |
| 2011 | 36,452 | 31.63 (31.30–31.96) | 15,000 | 21.23 (20.88–21.57) | 21,452 | 47.79 (47.15–48.44) |
| 2012 | 36,903 | 31.28 (30.96–31.60) | 14,995 | 20.80 (20.46–21.14) | 21,908 | 47.42 (46.79–48.05) |
| 2013 | 38,417 | 31.81 (31.49–32.13) | 15,288 | 20.77 (20.43–21.10) | 23,129 | 48.42 (47.79–49.05) |
| 2014 | 38,885 | 31.53 (31.21–31.84) | 15,537 | 20.77 (20.44–21.10) | 23,348 | 47.48 (46.86–48.09) |
| 2015 | 41,433 | 32.81 (32.49–33.13) | 16,357 | 21.45 (21.12–21.79) | 25,076 | 49.47 (48.86–50.09) |
| 2016 | 43,765 | 33.87 (33.55–34.19) | 17,319 | 22.31 (21.97–22.65) | 26,446 | 50.83 (50.21–51.44) |
| 2017 | 46,709 | 35.25 (34.93–35.57) | 18,263 | 23.09 (22.75–23.43) | 28,446 | 52.92 (52.30–53.54) |
| 2018 | 49,299 | 36.16 (35.84–36.48) | 19,237 | 23.77 (23.43–24.11) | 30,062 | 53.97 (53.35–54.59) |
| 2019 | 51,119 | 36.63 (36.31–36.95) | 19,605 | 23.79 (23.45–24.13) | 31,514 | 54.90 (54.29–55.51) |
| 2020 | 63,238 | 44.36 (44.01–44.71) | 24,415 | 29.16 (28.80–29.53) | 38,823 | 65.78 (65.12–66.44) |
| 2021 | 58,447 | 43.13 (42.78–43.49) | 22,334 | 28.37 (28.00–28.75) | 36,113 | 63.84 (63.17–64.51) |
| 2022 | 59,842 | 41.40 (41.07–41.73) | 22,567 | 26.67 (26.32–27.02) | 37,275 | 62.47 (61.83–63.11) |
| 2023 | 58,450 | 40.16 (39.84–40.49) | 22,077 | 26.26 (25.92–26.61) | 36,373 | 59.41 (58.79–60.03) |
| 2024 | 59,018 | 38.93 (38.62–39.25) | 22,005 | 25.21 (24.88–25.55) | 37,013 | 57.63 (57.04–58.23) |
| 2025 | 62,162 | 41.03 (40.70–41.35) | 22,983 | 26.37 (26.03–26.72) | 39,179 | 61.01 (60.40–61.63) |
| Total/Average | 1,149,731 | 34.99 (34.65–35.32) | 461,922 | 23.32 (22.97–23.68) | 687,809 | 52.91 (52.24–53.58) |

AAMRs are per 100,000 population and age-adjusted to the 2000 U.S. standard population. Parkinson’s disease-related deaths were identified from the CDC WONDER Multiple Cause of Death database using ICD-10 code G20, January 1999 through December 2025.

**Supplementary Table S3: Annual percent change (APC) and average annual percent change (AAPC) of Parkinson’s disease-related mortality among U.S. adults, 1999–2025.**

| Trend period | APC, % (95% CI) | p value |
| --- | --- | --- |
| **Overall — AAPC 0.56% (−0.10 to 1.22); p=0.099** |  |  |
| 1999–2014 | −0.65 (−1.16 to −0.14) | 0.015 |
| 2014–2021 | 4.70 (2.95 to 6.48) | <0.001 |
| 2021–2025 | −1.96 (−4.78 to 0.94) | 0.171 |
| **Female — AAPC 0.33% (−0.25 to 0.92); p=0.267** |  |  |
| 1999–2015 | −0.87 (−1.22 to −0.52) | <0.001 |
| 2015–2020 | 6.13 (3.43 to 8.90) | <0.001 |
| 2020–2025 | −1.41 (−3.00 to 0.21) | 0.085 |
| **Male — AAPC 0.40% (−0.25 to 1.05); p=0.225** |  |  |
| 1999–2014 | −0.83 (−1.34 to −0.32) | 0.003 |
| 2014–2021 | 4.51 (2.77 to 6.28) | <0.001 |
| 2021–2025 | −1.98 (−4.68 to 0.80) | 0.151 |
| **45–64 — AAPC 1.84% (0.34 to 3.35); p=0.016** |  |  |
| 1999–2003 | 4.75 (0.41 to 9.27) | 0.034 |
| 2003–2007 | −5.05 (−10.50 to 0.74) | 0.081 |
| 2007–2018 | 2.94 (2.11 to 3.78) | <0.001 |
| 2018–2021 | 9.99 (0.34 to 20.58) | 0.043 |
| 2021–2025 | −2.71 (−5.28 to −0.07) | 0.045 |
| **65–85+ — AAPC 0.60% (0.08 to 1.12); p=0.024** |  |  |
| 1999–2015 | −0.54 (−0.86 to −0.22) | 0.002 |
| 2015–2020 | 6.15 (3.76 to 8.61) | <0.001 |
| 2020–2025 | −1.12 (−2.53 to 0.30) | 0.114 |
| **White — AAPC 0.76% (0.13 to 1.40); p=0.018** |  |  |
| 1999–2014 | −0.52 (−1.00 to −0.04) | 0.035 |
| 2014–2021 | 4.91 (3.21 to 6.64) | <0.001 |
| 2021–2025 | −1.45 (−4.15 to 1.32) | 0.283 |
| **Hispanic or Latino — AAPC 0.64% (−0.67 to 1.98); p=0.339** |  |  |
| 1999–2017 | 0.51 (−0.02 to 1.04) | 0.057 |
| 2017–2020 | 9.32 (−2.23 to 22.24) | 0.111 |
| 2020–2025 | −3.76 (−5.89 to −1.59) | 0.002 |
| **American Indian or Alaska Native — AAPC −0.94% (−2.33 to 0.47); p=0.192** |  |  |
| 1999–2013 | −1.09 (−2.45 to 0.29) | 0.115 |
| 2013–2020 | 3.29 (−0.54 to 7.27) | 0.088 |
| 2020–2025 | −6.18 (−10.18 to −2.01) | 0.006 |
| **Asian or Pacific Islander — AAPC 0.37% (−0.40 to 1.15); p=0.343** |  |  |
| 1999–2013 | −1.08 (−1.94 to −0.21) | 0.018 |
| 2013–2021 | 4.86 (3.22 to 6.52) | <0.001 |
| 2021–2025 | −3.21 (−6.11 to −0.23) | 0.037 |
| **Black or African American — AAPC 0.93% (0.19 to 1.68); p=0.014** |  |  |
| 1999–2014 | −0.38 (−0.97 to 0.21) | 0.193 |
| 2014–2020 | 6.91 (4.27 to 9.63) | <0.001 |
| 2020–2025 | −2.02 (−4.11 to 0.11) | 0.061 |
| **Northeast — AAPC 0.57% (−0.08 to 1.23); p=0.083** |  |  |
| 1999–2011 | −0.91 (−2.03 to 0.23) | 0.113 |
| 2011–2025 | 1.86 (1.04 to 2.69) | <0.001 |
| **Midwest — AAPC 0.52% (−0.06 to 1.10); p=0.078** |  |  |
| 1999–2015 | −0.64 (−0.98 to −0.30) | <0.001 |
| 2015–2020 | 6.20 (3.54 to 8.93) | <0.001 |
| 2020–2025 | −1.26 (−2.83 to 0.33) | 0.113 |
| **South — AAPC 1.06% (0.41 to 1.72); p=0.001** |  |  |
| 1999–2014 | −0.33 (−0.86 to 0.20) | 0.206 |
| 2014–2021 | 5.66 (3.89 to 7.46) | <0.001 |
| 2021–2025 | −1.52 (−4.19 to 1.23) | 0.259 |
| **West — AAPC 0.07% (−0.47 to 0.61); p=0.800** |  |  |
| 1999–2014 | −1.01 (−1.42 to −0.59) | <0.001 |
| 2014–2021 | 4.05 (2.61 to 5.50) | <0.001 |
| 2021–2025 | −2.65 (−4.90 to −0.35) | 0.026 |
| **Metropolitan — AAPC 0.85% (0.27 to 1.44); p=0.004** (1999–2020) |  |  |
| 1999–2014 | −0.72 (−1.24 to −0.19) | 0.010 |
| 2014–2020 | 4.89 (3.09 to 6.73) | <0.001 |
| **Non-metropolitan — AAPC 1.28% (0.75 to 1.82); p<0.001** (1999–2020) |  |  |
| 1999–2015 | −0.40 (−0.81 to 0.01) | 0.054 |
| 2015–2020 | 6.87 (4.76 to 9.03) | <0.001 |

APC, annual percent change within a joinpoint segment; AAPC, average annual percent change; CI, confidence interval. Trends are from the NCI Joinpoint Regression Program on log-transformed age-adjusted rates. Urbanization segments cover 1999–2020. ICD-10 G20, CDC WONDER Multiple Cause of Death. ᶜ The Hispanic or Latino stratum was refitted on a directly queried Hispanic-origin series.

**Supplementary Table S4.** Projected age-adjusted Parkinson’s disease mortality rates with 95% prediction intervals, 2026–2030, by stratum and specification.

| Stratum | Specification | Year | Projected AAMR (95% PI) |
| --- | --- | --- | --- |
| Overall | Primary (full 1999–2025) | 2026 | 41.14 (37.51 to 45.12) |
| Overall | Primary (full 1999–2025) | 2027 | 41.29 (36.26 to 47.01) |
| Overall | Primary (full 1999–2025) | 2028 | 41.44 (35.35 to 48.57) |
| Overall | Primary (full 1999–2025) | 2029 | 41.58 (34.61 to 49.97) |
| Overall | Primary (full 1999–2025) | 2030 | 41.73 (33.97 to 51.27) |
| Overall | Sensitivity (pre-pandemic anchor) | 2020 | 37.02 (35.27 to 38.87) |
| Overall | Sensitivity (pre-pandemic anchor) | 2021 | 37.25 (34.73 to 39.96) |
| Overall | Sensitivity (pre-pandemic anchor) | 2022 | 37.42 (33.84 to 41.38) |
| Overall | Sensitivity (pre-pandemic anchor) | 2023 | 37.57 (33.13 to 42.61) |
| Overall | Sensitivity (pre-pandemic anchor) | 2024 | 37.71 (32.50 to 43.74) |
| Overall | Sensitivity (pre-pandemic anchor) | 2025 | 37.82 (31.93 to 44.80) |
| Overall | Sensitivity (pre-pandemic anchor) | 2026 | 37.93 (31.40 to 45.81) |
| Overall | Sensitivity (pre-pandemic anchor) | 2027 | 38.02 (30.90 to 46.78) |
| Overall | Sensitivity (pre-pandemic anchor) | 2028 | 38.11 (30.44 to 47.71) |
| Overall | Sensitivity (pre-pandemic anchor) | 2029 | 38.19 (30.00 to 48.62) |
| Overall | Sensitivity (pre-pandemic anchor) | 2030 | 38.27 (29.59 to 49.50) |
| Female | Primary (full 1999–2025) | 2026 | 26.37 (23.84 to 29.16) |
| Female | Primary (full 1999–2025) | 2027 | 26.40 (22.96 to 30.36) |
| Female | Primary (full 1999–2025) | 2028 | 26.44 (22.30 to 31.35) |
| Female | Primary (full 1999–2025) | 2029 | 26.48 (21.76 to 32.21) |
| Female | Primary (full 1999–2025) | 2030 | 26.52 (21.30 to 33.01) |
| Female | Sensitivity (pre-pandemic anchor) | 2020 | 23.71 (22.30 to 25.20) |
| Female | Sensitivity (pre-pandemic anchor) | 2021 | 23.64 (21.76 to 25.69) |
| Female | Sensitivity (pre-pandemic anchor) | 2022 | 23.60 (21.37 to 26.06) |
| Female | Sensitivity (pre-pandemic anchor) | 2023 | 23.56 (21.06 to 26.36) |
| Female | Sensitivity (pre-pandemic anchor) | 2024 | 23.54 (20.81 to 26.63) |
| Female | Sensitivity (pre-pandemic anchor) | 2025 | 23.53 (20.59 to 26.88) |
| Female | Sensitivity (pre-pandemic anchor) | 2026 | 23.52 (20.40 to 27.11) |
| Female | Sensitivity (pre-pandemic anchor) | 2027 | 23.52 (20.24 to 27.33) |
| Female | Sensitivity (pre-pandemic anchor) | 2028 | 23.52 (20.09 to 27.55) |
| Female | Sensitivity (pre-pandemic anchor) | 2029 | 23.53 (19.95 to 27.75) |
| Female | Sensitivity (pre-pandemic anchor) | 2030 | 23.54 (19.83 to 27.95) |
| Male | Primary (full 1999–2025) | 2026 | 61.09 (55.90 to 66.76) |
| Male | Primary (full 1999–2025) | 2027 | 61.20 (53.99 to 69.38) |
| Male | Primary (full 1999–2025) | 2028 | 61.31 (52.56 to 71.52) |
| Male | Primary (full 1999–2025) | 2029 | 61.42 (51.38 to 73.42) |
| Male | Primary (full 1999–2025) | 2030 | 61.53 (50.37 to 75.16) |
| Male | Sensitivity (pre-pandemic anchor) | 2020 | 55.57 (52.72 to 58.58) |
| Male | Sensitivity (pre-pandemic anchor) | 2021 | 56.19 (51.95 to 60.78) |
| Male | Sensitivity (pre-pandemic anchor) | 2022 | 56.69 (50.58 to 63.55) |
| Male | Sensitivity (pre-pandemic anchor) | 2023 | 57.17 (49.31 to 66.29) |
| Male | Sensitivity (pre-pandemic anchor) | 2024 | 57.62 (48.06 to 69.09) |
| Male | Sensitivity (pre-pandemic anchor) | 2025 | 58.06 (46.82 to 71.99) |
| Male | Sensitivity (pre-pandemic anchor) | 2026 | 58.48 (45.58 to 75.02) |
| Male | Sensitivity (pre-pandemic anchor) | 2027 | 58.89 (44.34 to 78.21) |
| Male | Sensitivity (pre-pandemic anchor) | 2028 | 59.29 (43.09 to 81.58) |
| Male | Sensitivity (pre-pandemic anchor) | 2029 | 59.69 (41.85 to 85.13) |
| Male | Sensitivity (pre-pandemic anchor) | 2030 | 60.08 (40.60 to 88.90) |
| 45–64 | Primary (full 1999–2025) | 2026 | 1.53 (1.34 to 1.74) |
| 45–64 | Primary (full 1999–2025) | 2027 | 1.53 (1.27 to 1.85) |
| 45–64 | Primary (full 1999–2025) | 2028 | 1.53 (1.21 to 1.94) |
| 45–64 | Primary (full 1999–2025) | 2029 | 1.54 (1.16 to 2.04) |
| 45–64 | Primary (full 1999–2025) | 2030 | 1.54 (1.12 to 2.14) |
| 45–64 | Sensitivity (pre-pandemic anchor) | 2020 | 1.37 (1.21 to 1.54) |
| 45–64 | Sensitivity (pre-pandemic anchor) | 2021 | 1.38 (1.17 to 1.62) |
| 45–64 | Sensitivity (pre-pandemic anchor) | 2022 | 1.39 (1.14 to 1.69) |
| 45–64 | Sensitivity (pre-pandemic anchor) | 2023 | 1.40 (1.11 to 1.76) |
| 45–64 | Sensitivity (pre-pandemic anchor) | 2024 | 1.41 (1.09 to 1.83) |
| 45–64 | Sensitivity (pre-pandemic anchor) | 2025 | 1.43 (1.07 to 1.89) |
| 45–64 | Sensitivity (pre-pandemic anchor) | 2026 | 1.44 (1.06 to 1.96) |
| 45–64 | Sensitivity (pre-pandemic anchor) | 2027 | 1.45 (1.04 to 2.02) |
| 45–64 | Sensitivity (pre-pandemic anchor) | 2028 | 1.46 (1.02 to 2.08) |
| 45–64 | Sensitivity (pre-pandemic anchor) | 2029 | 1.47 (1.01 to 2.14) |
| 45–64 | Sensitivity (pre-pandemic anchor) | 2030 | 1.48 (1.00 to 2.21) |
| ≥65 | Primary (full 1999–2025) | 2026 | 110.73 (100.95 to 121.45) |
| ≥65 | Primary (full 1999–2025) | 2027 | 111.12 (97.59 to 126.51) |
| ≥65 | Primary (full 1999–2025) | 2028 | 111.50 (95.13 to 130.70) |
| ≥65 | Primary (full 1999–2025) | 2029 | 111.89 (93.12 to 134.44) |
| ≥65 | Primary (full 1999–2025) | 2030 | 112.27 (91.40 to 137.91) |
| ≥65 | Sensitivity (pre-pandemic anchor) | 2020 | 100.58 (95.66 to 105.76) |
| ≥65 | Sensitivity (pre-pandemic anchor) | 2021 | 101.75 (94.22 to 109.87) |
| ≥65 | Sensitivity (pre-pandemic anchor) | 2022 | 102.77 (91.56 to 115.34) |
| ≥65 | Sensitivity (pre-pandemic anchor) | 2023 | 103.74 (89.13 to 120.73) |
| ≥65 | Sensitivity (pre-pandemic anchor) | 2024 | 104.66 (86.76 to 126.26) |
| ≥65 | Sensitivity (pre-pandemic anchor) | 2025 | 105.55 (84.39 to 132.02) |
| ≥65 | Sensitivity (pre-pandemic anchor) | 2026 | 106.42 (82.02 to 138.07) |
| ≥65 | Sensitivity (pre-pandemic anchor) | 2027 | 107.26 (79.64 to 144.46) |
| ≥65 | Sensitivity (pre-pandemic anchor) | 2028 | 108.09 (77.26 to 151.23) |
| ≥65 | Sensitivity (pre-pandemic anchor) | 2029 | 108.91 (74.88 to 158.41) |
| ≥65 | Sensitivity (pre-pandemic anchor) | 2030 | 109.72 (72.50 to 166.05) |
| White, non-Hispanic | Primary (full 1999–2025) | 2026 | 46.13 (42.16 to 50.47) |
| White, non-Hispanic | Primary (full 1999–2025) | 2027 | 46.35 (40.79 to 52.66) |
| White, non-Hispanic | Primary (full 1999–2025) | 2028 | 46.57 (39.80 to 54.49) |
| White, non-Hispanic | Primary (full 1999–2025) | 2029 | 46.78 (38.99 to 56.14) |
| White, non-Hispanic | Primary (full 1999–2025) | 2030 | 47.00 (38.30 to 57.68) |
| White, non-Hispanic | Sensitivity (pre-pandemic anchor) | 2020 | 40.56 (38.61 to 42.60) |
| White, non-Hispanic | Sensitivity (pre-pandemic anchor) | 2021 | 40.99 (38.26 to 43.91) |
| White, non-Hispanic | Sensitivity (pre-pandemic anchor) | 2022 | 41.21 (37.31 to 45.50) |
| White, non-Hispanic | Sensitivity (pre-pandemic anchor) | 2023 | 41.39 (36.54 to 46.89) |
| White, non-Hispanic | Sensitivity (pre-pandemic anchor) | 2024 | 41.56 (35.86 to 48.16) |
| White, non-Hispanic | Sensitivity (pre-pandemic anchor) | 2025 | 41.71 (35.25 to 49.36) |
| White, non-Hispanic | Sensitivity (pre-pandemic anchor) | 2026 | 41.85 (34.67 to 50.50) |
| White, non-Hispanic | Sensitivity (pre-pandemic anchor) | 2027 | 41.97 (34.14 to 51.60) |
| White, non-Hispanic | Sensitivity (pre-pandemic anchor) | 2028 | 42.09 (33.63 to 52.66) |
| White, non-Hispanic | Sensitivity (pre-pandemic anchor) | 2029 | 42.20 (33.16 to 53.70) |
| White, non-Hispanic | Sensitivity (pre-pandemic anchor) | 2030 | 42.30 (32.71 to 54.70) |
| Hispanic or Latino | Primary (full 1999–2025) | 2026 | 28.55 (24.56 to 33.18) |
| Hispanic or Latino | Primary (full 1999–2025) | 2027 | 28.82 (24.39 to 34.05) |
| Hispanic or Latino | Primary (full 1999–2025) | 2028 | 28.95 (23.98 to 34.94) |
| Hispanic or Latino | Primary (full 1999–2025) | 2029 | 29.12 (23.70 to 35.78) |
| Hispanic or Latino | Primary (full 1999–2025) | 2030 | 29.28 (23.43 to 36.60) |
| Hispanic or Latino | Sensitivity (pre-pandemic anchor) | 2020 | 26.56 (23.93 to 29.48) |
| Hispanic or Latino | Sensitivity (pre-pandemic anchor) | 2021 | 26.84 (23.99 to 30.03) |
| Hispanic or Latino | Sensitivity (pre-pandemic anchor) | 2022 | 27.13 (24.03 to 30.62) |
| Hispanic or Latino | Sensitivity (pre-pandemic anchor) | 2023 | 27.42 (24.06 to 31.26) |
| Hispanic or Latino | Sensitivity (pre-pandemic anchor) | 2024 | 27.73 (24.06 to 31.96) |
| Hispanic or Latino | Sensitivity (pre-pandemic anchor) | 2025 | 28.05 (24.04 to 32.73) |
| Hispanic or Latino | Sensitivity (pre-pandemic anchor) | 2026 | 28.38 (24.00 to 33.55) |
| Hispanic or Latino | Sensitivity (pre-pandemic anchor) | 2027 | 28.72 (23.94 to 34.44) |
| Hispanic or Latino | Sensitivity (pre-pandemic anchor) | 2028 | 29.07 (23.87 to 35.40) |
| Hispanic or Latino | Sensitivity (pre-pandemic anchor) | 2029 | 29.43 (23.78 to 36.43) |
| Hispanic or Latino | Sensitivity (pre-pandemic anchor) | 2030 | 29.81 (23.67 to 37.54) |
| American Indian or Alaska Native, non-Hispanic | Primary (full 1999–2025) | 2026 | 23.19 (19.22 to 27.98) |
| American Indian or Alaska Native, non-Hispanic | Primary (full 1999–2025) | 2027 | 23.52 (19.33 to 28.62) |
| American Indian or Alaska Native, non-Hispanic | Primary (full 1999–2025) | 2028 | 23.50 (19.31 to 28.60) |
| American Indian or Alaska Native, non-Hispanic | Primary (full 1999–2025) | 2029 | 23.48 (19.30 to 28.58) |
| American Indian or Alaska Native, non-Hispanic | Primary (full 1999–2025) | 2030 | 23.47 (19.28 to 28.56) |
| American Indian or Alaska Native, non-Hispanic | Sensitivity (pre-pandemic anchor) | 2020 | 23.89 (20.21 to 28.22) |
| American Indian or Alaska Native, non-Hispanic | Sensitivity (pre-pandemic anchor) | 2021 | 23.85 (20.05 to 28.36) |
| American Indian or Alaska Native, non-Hispanic | Sensitivity (pre-pandemic anchor) | 2022 | 23.84 (20.05 to 28.36) |
| American Indian or Alaska Native, non-Hispanic | Sensitivity (pre-pandemic anchor) | 2023 | 23.84 (20.04 to 28.36) |
| American Indian or Alaska Native, non-Hispanic | Sensitivity (pre-pandemic anchor) | 2024 | 23.84 (20.04 to 28.35) |
| American Indian or Alaska Native, non-Hispanic | Sensitivity (pre-pandemic anchor) | 2025 | 23.83 (20.04 to 28.35) |
| American Indian or Alaska Native, non-Hispanic | Sensitivity (pre-pandemic anchor) | 2026 | 23.83 (20.04 to 28.35) |
| American Indian or Alaska Native, non-Hispanic | Sensitivity (pre-pandemic anchor) | 2027 | 23.83 (20.03 to 28.34) |
| American Indian or Alaska Native, non-Hispanic | Sensitivity (pre-pandemic anchor) | 2028 | 23.83 (20.03 to 28.34) |
| American Indian or Alaska Native, non-Hispanic | Sensitivity (pre-pandemic anchor) | 2029 | 23.82 (20.03 to 28.34) |
| American Indian or Alaska Native, non-Hispanic | Sensitivity (pre-pandemic anchor) | 2030 | 23.82 (20.03 to 28.34) |
| Asian or Pacific Islander, non-Hispanic | Primary (full 1999–2025) | 2026 | 24.95 (22.06 to 28.22) |
| Asian or Pacific Islander, non-Hispanic | Primary (full 1999–2025) | 2027 | 25.23 (21.52 to 29.57) |
| Asian or Pacific Islander, non-Hispanic | Primary (full 1999–2025) | 2028 | 25.06 (20.65 to 30.41) |
| Asian or Pacific Islander, non-Hispanic | Primary (full 1999–2025) | 2029 | 25.32 (20.35 to 31.52) |
| Asian or Pacific Islander, non-Hispanic | Primary (full 1999–2025) | 2030 | 25.17 (19.67 to 32.21) |
| Asian or Pacific Islander, non-Hispanic | Sensitivity (pre-pandemic anchor) | 2020 | 24.42 (21.48 to 27.77) |
| Asian or Pacific Islander, non-Hispanic | Sensitivity (pre-pandemic anchor) | 2021 | 25.52 (21.91 to 29.73) |
| Asian or Pacific Islander, non-Hispanic | Sensitivity (pre-pandemic anchor) | 2022 | 25.67 (20.41 to 32.29) |
| Asian or Pacific Islander, non-Hispanic | Sensitivity (pre-pandemic anchor) | 2023 | 26.75 (20.69 to 34.59) |
| Asian or Pacific Islander, non-Hispanic | Sensitivity (pre-pandemic anchor) | 2024 | 26.85 (19.08 to 37.78) |
| Asian or Pacific Islander, non-Hispanic | Sensitivity (pre-pandemic anchor) | 2025 | 27.91 (19.19 to 40.60) |
| Asian or Pacific Islander, non-Hispanic | Sensitivity (pre-pandemic anchor) | 2026 | 27.95 (17.59 to 44.42) |
| Asian or Pacific Islander, non-Hispanic | Sensitivity (pre-pandemic anchor) | 2027 | 29.02 (17.57 to 47.92) |
| Asian or Pacific Islander, non-Hispanic | Sensitivity (pre-pandemic anchor) | 2028 | 29.02 (16.04 to 52.51) |
| Asian or Pacific Islander, non-Hispanic | Sensitivity (pre-pandemic anchor) | 2029 | 30.09 (15.92 to 56.88) |
| Asian or Pacific Islander, non-Hispanic | Sensitivity (pre-pandemic anchor) | 2030 | 30.06 (14.48 to 62.42) |
| Black or African American, non-Hispanic | Primary (full 1999–2025) | 2026 | 23.16 (20.27 to 26.46) |
| Black or African American, non-Hispanic | Primary (full 1999–2025) | 2027 | 23.25 (19.42 to 27.83) |
| Black or African American, non-Hispanic | Primary (full 1999–2025) | 2028 | 23.33 (18.75 to 29.03) |
| Black or African American, non-Hispanic | Primary (full 1999–2025) | 2029 | 23.42 (18.18 to 30.16) |
| Black or African American, non-Hispanic | Primary (full 1999–2025) | 2030 | 23.50 (17.68 to 31.24) |
| Black or African American, non-Hispanic | Sensitivity (pre-pandemic anchor) | 2020 | 20.66 (18.94 to 22.53) |
| Black or African American, non-Hispanic | Sensitivity (pre-pandemic anchor) | 2021 | 20.76 (18.39 to 23.44) |
| Black or African American, non-Hispanic | Sensitivity (pre-pandemic anchor) | 2022 | 20.86 (17.98 to 24.21) |
| Black or African American, non-Hispanic | Sensitivity (pre-pandemic anchor) | 2023 | 20.97 (17.65 to 24.91) |
| Black or African American, non-Hispanic | Sensitivity (pre-pandemic anchor) | 2024 | 21.07 (17.37 to 25.56) |
| Black or African American, non-Hispanic | Sensitivity (pre-pandemic anchor) | 2025 | 21.17 (17.12 to 26.18) |
| Black or African American, non-Hispanic | Sensitivity (pre-pandemic anchor) | 2026 | 21.27 (16.90 to 26.78) |
| Black or African American, non-Hispanic | Sensitivity (pre-pandemic anchor) | 2027 | 21.37 (16.69 to 27.37) |
| Black or African American, non-Hispanic | Sensitivity (pre-pandemic anchor) | 2028 | 21.47 (16.50 to 27.94) |
| Black or African American, non-Hispanic | Sensitivity (pre-pandemic anchor) | 2029 | 21.58 (16.33 to 28.51) |
| Black or African American, non-Hispanic | Sensitivity (pre-pandemic anchor) | 2030 | 21.68 (16.16 to 29.07) |
| Northeast | Primary (full 1999–2025) | 2026 | 34.74 (30.93 to 39.01) |
| Northeast | Primary (full 1999–2025) | 2027 | 34.47 (29.80 to 39.86) |
| Northeast | Primary (full 1999–2025) | 2028 | 34.28 (29.01 to 40.52) |
| Northeast | Primary (full 1999–2025) | 2029 | 34.16 (28.41 to 41.08) |
| Northeast | Primary (full 1999–2025) | 2030 | 34.08 (27.93 to 41.59) |
| Northeast | Sensitivity (pre-pandemic anchor) | 2020 | 31.97 (29.50 to 34.65) |
| Northeast | Sensitivity (pre-pandemic anchor) | 2021 | 32.04 (28.78 to 35.67) |
| Northeast | Sensitivity (pre-pandemic anchor) | 2022 | 32.15 (28.19 to 36.66) |
| Northeast | Sensitivity (pre-pandemic anchor) | 2023 | 32.28 (27.64 to 37.70) |
| Northeast | Sensitivity (pre-pandemic anchor) | 2024 | 32.43 (27.11 to 38.80) |
| Northeast | Sensitivity (pre-pandemic anchor) | 2025 | 32.59 (26.57 to 39.97) |
| Northeast | Sensitivity (pre-pandemic anchor) | 2026 | 32.75 (26.02 to 41.22) |
| Northeast | Sensitivity (pre-pandemic anchor) | 2027 | 32.91 (25.45 to 42.55) |
| Northeast | Sensitivity (pre-pandemic anchor) | 2028 | 33.07 (24.87 to 43.98) |
| Northeast | Sensitivity (pre-pandemic anchor) | 2029 | 33.24 (24.27 to 45.51) |
| Northeast | Sensitivity (pre-pandemic anchor) | 2030 | 33.40 (23.66 to 47.14) |
| Midwest | Primary (full 1999–2025) | 2026 | 44.36 (39.91 to 49.30) |
| Midwest | Primary (full 1999–2025) | 2027 | 44.48 (38.51 to 51.38) |
| Midwest | Primary (full 1999–2025) | 2028 | 44.61 (37.46 to 53.11) |
| Midwest | Primary (full 1999–2025) | 2029 | 44.73 (36.60 to 54.66) |
| Midwest | Primary (full 1999–2025) | 2030 | 44.85 (35.86 to 56.09) |
| Midwest | Sensitivity (pre-pandemic anchor) | 2020 | 39.94 (37.56 to 42.48) |
| Midwest | Sensitivity (pre-pandemic anchor) | 2021 | 40.21 (36.81 to 43.93) |
| Midwest | Sensitivity (pre-pandemic anchor) | 2022 | 40.43 (36.14 to 45.23) |
| Midwest | Sensitivity (pre-pandemic anchor) | 2023 | 40.62 (35.53 to 46.44) |
| Midwest | Sensitivity (pre-pandemic anchor) | 2024 | 40.78 (34.97 to 47.56) |
| Midwest | Sensitivity (pre-pandemic anchor) | 2025 | 40.92 (34.43 to 48.62) |
| Midwest | Sensitivity (pre-pandemic anchor) | 2026 | 41.04 (33.93 to 49.63) |
| Midwest | Sensitivity (pre-pandemic anchor) | 2027 | 41.14 (33.46 to 50.58) |
| Midwest | Sensitivity (pre-pandemic anchor) | 2028 | 41.23 (33.01 to 51.50) |
| Midwest | Sensitivity (pre-pandemic anchor) | 2029 | 41.31 (32.58 to 52.39) |
| Midwest | Sensitivity (pre-pandemic anchor) | 2030 | 41.39 (32.17 to 53.24) |
| South | Primary (full 1999–2025) | 2026 | 43.44 (39.40 to 47.89) |
| South | Primary (full 1999–2025) | 2027 | 44.22 (38.48 to 50.82) |
| South | Primary (full 1999–2025) | 2028 | 45.03 (37.89 to 53.51) |
| South | Primary (full 1999–2025) | 2029 | 45.87 (37.46 to 56.17) |
| South | Primary (full 1999–2025) | 2030 | 46.74 (37.11 to 58.86) |
| South | Sensitivity (pre-pandemic anchor) | 2020 | 37.12 (34.83 to 39.56) |
| South | Sensitivity (pre-pandemic anchor) | 2021 | 37.39 (34.17 to 40.92) |
| South | Sensitivity (pre-pandemic anchor) | 2022 | 37.65 (33.18 to 42.71) |
| South | Sensitivity (pre-pandemic anchor) | 2023 | 37.87 (32.38 to 44.29) |
| South | Sensitivity (pre-pandemic anchor) | 2024 | 38.08 (31.67 to 45.78) |
| South | Sensitivity (pre-pandemic anchor) | 2025 | 38.27 (31.03 to 47.20) |
| South | Sensitivity (pre-pandemic anchor) | 2026 | 38.44 (30.43 to 48.57) |
| South | Sensitivity (pre-pandemic anchor) | 2027 | 38.61 (29.87 to 49.91) |
| South | Sensitivity (pre-pandemic anchor) | 2028 | 38.77 (29.34 to 51.22) |
| South | Sensitivity (pre-pandemic anchor) | 2029 | 38.92 (28.84 to 52.51) |
| South | Sensitivity (pre-pandemic anchor) | 2030 | 39.06 (28.37 to 53.78) |
| West | Primary (full 1999–2025) | 2026 | 40.08 (37.20 to 43.18) |
| West | Primary (full 1999–2025) | 2027 | 39.73 (35.74 to 44.17) |
| West | Primary (full 1999–2025) | 2028 | 39.23 (34.15 to 45.07) |
| West | Primary (full 1999–2025) | 2029 | 38.92 (32.99 to 45.91) |
| West | Primary (full 1999–2025) | 2030 | 38.96 (32.63 to 46.51) |
| West | Sensitivity (pre-pandemic anchor) | 2020 | 37.72 (35.68 to 39.86) |
| West | Sensitivity (pre-pandemic anchor) | 2021 | 37.73 (34.92 to 40.76) |
| West | Sensitivity (pre-pandemic anchor) | 2022 | 37.74 (34.34 to 41.47) |
| West | Sensitivity (pre-pandemic anchor) | 2023 | 37.75 (33.85 to 42.09) |
| West | Sensitivity (pre-pandemic anchor) | 2024 | 37.76 (33.43 to 42.64) |
| West | Sensitivity (pre-pandemic anchor) | 2025 | 37.77 (33.06 to 43.15) |
| West | Sensitivity (pre-pandemic anchor) | 2026 | 37.78 (32.72 to 43.62) |
| West | Sensitivity (pre-pandemic anchor) | 2027 | 37.79 (32.41 to 44.07) |
| West | Sensitivity (pre-pandemic anchor) | 2028 | 37.80 (32.12 to 44.49) |
| West | Sensitivity (pre-pandemic anchor) | 2029 | 37.81 (31.85 to 44.90) |
| West | Sensitivity (pre-pandemic anchor) | 2030 | 37.83 (31.59 to 45.29) |

AAMR, age-adjusted mortality rate per 100,000 (2000 U.S. standard population); PI, prediction interval. All models were fitted to the natural logarithm of the AAMR and back-transformed, so intervals are strictly positive and asymmetric. Intervals combine within-model forecast variance and between-model dispersion.

**Supplementary Table S5: Trends in Age-Adjusted Mortality Rates and Deaths from Parkinson’s Disease among United States Adults, stratified by Age Group, 1999–2025.**

| **Year** | **45–64 years** | **45–64 years** | **65–85+ years** | **65–85+ years** |
| --- | --- | --- | --- | --- |
| **Year** | **Deaths** | **AAMR (95% CI)** | **Deaths** | **AAMR (95% CI)** |
| 1999 | 582 | 0.95 (0.87–1.02) | 30,428 | 88.62 (87.62–89.61) |
| 2000 | 623 | 0.99 (0.91–1.06) | 31,670 | 91.07 (90.07–92.07) |
| 2001 | 685 | 1.06 (0.98–1.14) | 32,186 | 91.13 (90.13–92.12) |
| 2002 | 711 | 1.09 (1.01–1.17) | 32,816 | 91.86 (90.86–92.85) |
| 2003 | 792 | 1.13 (1.05–1.20) | 33,561 | 92.58 (91.59–93.57) |
| 2004 | 769 | 1.05 (0.97–1.12) | 32,947 | 89.83 (88.86–90.80) |
| 2005 | 860 | 1.13 (1.05–1.20) | 35,001 | 93.82 (92.84–94.81) |
| 2006 | 782 | 0.95 (0.88–1.01) | 33,483 | 88.19 (87.25–89.14) |
| 2007 | 804 | 0.95 (0.88–1.01) | 33,639 | 87.15 (86.22–88.08) |
| 2008 | 800 | 0.91 (0.84–0.97) | 33,512 | 85.33 (84.42–86.25) |
| 2009 | 878 | 0.99 (0.92–1.05) | 32,888 | 82.43 (81.53–83.32) |
| 2010 | 906 | 1.05 (0.98–1.12) | 34,269 | 84.68 (83.78–85.58) |
| 2011 | 971 | 1.05 (0.98–1.11) | 35,481 | 85.38 (84.48–86.27) |
| 2012 | 1,020 | 1.09 (1.02–1.15) | 35,883 | 84.33 (83.45–85.21) |
| 2013 | 1,073 | 1.13 (1.06–1.19) | 37,344 | 85.72 (84.85–86.60) |
| 2014 | 1,019 | 1.09 (1.02–1.15) | 37,866 | 85.01 (84.15–85.88) |
| 2015 | 1,129 | 1.16 (1.10–1.23) | 40,304 | 88.42 (87.55–89.29) |
| 2016 | 1,221 | 1.20 (1.14–1.27) | 42,544 | 91.26 (90.39–92.14) |
| 2017 | 1,293 | 1.28 (1.21–1.35) | 45,416 | 94.94 (94.06–95.82) |
| 2018 | 1,335 | 1.28 (1.21–1.35) | 47,964 | 97.45 (96.57–98.33) |
| 2019 | 1,405 | 1.36 (1.29–1.43) | 49,714 | 98.62 (97.75–99.49) |
| 2020 | 1,700 | 1.64 (1.56–1.71) | 61,538 | 119.44 (118.49–120.39) |
| 2021 | 1,661 | 1.62 (1.54–1.70) | 56,786 | 116.09 (115.13–117.05) |
| 2022 | 1,690 | 1.70 (1.62–1.78) | 58,152 | 111.17 (110.26–112.08) |
| 2023 | 1,602 | 1.62 (1.54–1.70) | 56,848 | 107.89 (107.00–108.79) |
| 2024 | 1,556 | 1.56 (1.48–1.64) | 57,462 | 104.61 (103.76–105.48) |
| 2025 | 1,535 | 1.52 (1.44–1.60) | 60,627 | 110.45 (109.57–111.34) |
| Total/Average | 29,402 | 1.21 (1.13–1.28) | 1,120,329 | 94.35 (93.43–95.27) |

Age-specific rates are age-adjusted within each age band. Parkinson’s disease-related deaths were identified using ICD-10 code G20. AAMRs are expressed per 100,000.

**Supplementary Table S6: Trends in Age-Adjusted Mortality Rates and Deaths from Parkinson’s Disease among United States Adults, stratified by Race and Ethnicity, 1999–2025.**

| **Year** | **White** | **White** | **Black or African American** | **Black or African American** | **Hispanic or Latino** | **Hispanic or Latino** | **Asian or Pacific Islander** | **Asian or Pacific Islander** | **American Indian or Alaska Native** | **American Indian or Alaska Native** |
| --- | --- | --- | --- | --- | --- | --- | --- | --- | --- | --- |
| **Year** | **Deaths** | **AAMR (95% CI)** | **Deaths** | **AAMR (95% CI)** | **Deaths** | **AAMR (95% CI)** | **Deaths** | **AAMR (95% CI)** | **Deaths** | **AAMR (95% CI)** |
| 1999 | 28,501 | 34.97 (34.56–35.37) | 1,219 | 16.81 (15.87–17.76) | 846 | 22.28 (20.75–23.80) | 373 | 21.14 (18.94–23.34) | 71 | 23.68 (18.42–29.97) |
| 2000 | 29,673 | 36.04 (35.63–36.45) | 1,246 | 17.01 (16.06–17.96) | 918 | 22.82 (21.32–24.32) | 385 | 20.56 (18.47–22.66) | 71 | 21.28 (16.55–26.93) |
| 2001 | 30,031 | 36.02 (35.61–36.43) | 1,249 | 16.80 (15.87–17.74) | 1,063 | 24.61 (23.10–26.11) | 450 | 21.41 (19.39–23.43) | 78 | 23.27 (18.30–29.17) |
| 2002 | 30,653 | 36.45 (36.04–36.85) | 1,303 | 17.32 (16.38–18.27) | 1,020 | 22.50 (21.09–23.90) | 456 | 20.33 (18.43–22.24) | 95 | 27.38 (22.05–33.62) |
| 2003 | 31,251 | 36.72 (36.32–37.13) | 1,379 | 18.07 (17.11–19.03) | 1,092 | 23.13 (21.74–24.53) | 530 | 22.63 (20.67–24.60) | 101 | 28.74 (23.01–34.48) |
| 2004 | 30,580 | 35.62 (35.22–36.02) | 1,383 | 17.92 (16.97–18.87) | 1,140 | 22.71 (21.37–24.05) | 533 | 21.25 (19.42–23.09) | 80 | 22.31 (17.60–27.88) |
| 2005 | 32,389 | 37.22 (36.81–37.62) | 1,428 | 18.11 (17.16–19.05) | 1,371 | 25.84 (24.45–27.23) | 570 | 21.13 (19.37–22.89) | 103 | 25.96 (20.77–31.15) |
| 2006 | 30,871 | 35.00 (34.61–35.39) | 1,327 | 16.50 (15.61–17.39) | 1,370 | 24.73 (23.41–26.06) | 596 | 20.97 (19.27–22.68) | 101 | 26.29 (21.03–31.55) |
| 2007 | 30,928 | 34.62 (34.23–35.01) | 1,433 | 17.46 (16.55–18.37) | 1,364 | 23.09 (21.85–24.33) | 622 | 20.66 (19.02–22.30) | 96 | 24.22 (19.50–29.73) |
| 2008 | 30,765 | 34.01 (33.63–34.39) | 1,380 | 16.35 (15.48–17.22) | 1,471 | 23.83 (22.60–25.06) | 604 | 18.58 (17.09–20.08) | 92 | 22.78 (18.29–28.03) |
| 2009 | 30,242 | 33.00 (32.62–33.37) | 1,327 | 15.37 (14.53–16.20) | 1,445 | 22.17 (21.01–23.32) | 651 | 19.12 (17.64–20.60) | 101 | 22.37 (17.87–26.88) |
| 2010 | 31,375 | 33.90 (33.52–34.28) | 1,414 | 15.95 (15.11–16.79) | 1,639 | 24.27 (23.08–25.45) | 648 | 18.13 (16.72–19.54) | 99 | 21.90 (17.70–26.80) |
| 2011 | 32,324 | 34.24 (33.86–34.62) | 1,500 | 16.21 (15.38–17.04) | 1,709 | 23.20 (22.09–24.31) | 805 | 20.89 (19.44–22.35) | 114 | 23.12 (18.76–27.48) |
| 2012 | 32,659 | 34.03 (33.66–34.40) | 1,559 | 16.30 (15.48–17.12) | 1,803 | 23.24 (22.16–24.32) | 767 | 18.49 (17.17–19.81) | 115 | 22.74 (18.50–26.99) |
| 2013 | 33,677 | 34.47 (34.10–34.84) | 1,693 | 17.19 (16.37–18.02) | 2,070 | 24.91 (23.83–25.99) | 866 | 19.39 (18.09–20.69) | 111 | 20.51 (16.61–24.42) |
| 2014 | 33,953 | 34.31 (33.94–34.68) | 1,752 | 17.14 (16.33–17.96) | 2,101 | 23.73 (22.70–24.75) | 949 | 19.57 (18.32–20.83) | 130 | 22.51 (18.55–26.46) |
| 2015 | 35,892 | 35.70 (35.32–36.07) | 1,885 | 17.75 (16.93–18.56) | 2,394 | 25.37 (24.35–26.40) | 1,119 | 21.39 (20.12–22.65) | 143 | 23.35 (19.44–27.27) |
| 2016 | 37,872 | 37.06 (36.68–37.43) | 2,079 | 18.93 (18.11–19.76) | 2,526 | 25.50 (24.49–26.51) | 1,141 | 20.72 (19.51–21.93) | 147 | 22.80 (19.03–26.56) |
| 2017 | 40,196 | 38.58 (38.20–38.96) | 2,267 | 19.88 (19.05–20.71) | 2,761 | 26.36 (25.36–27.35) | 1,309 | 22.10 (20.90–23.31) | 176 | 26.34 (22.39–30.29) |
| 2018 | 42,166 | 39.49 (39.12–39.87) | 2,503 | 21.22 (20.37–22.06) | 2,982 | 27.20 (26.21–28.19) | 1,451 | 23.32 (22.11–24.53) | 197 | 27.32 (23.44–31.20) |
| 2019 | 43,685 | 40.24 (39.86–40.62) | 2,522 | 20.53 (19.72–21.34) | 3,114 | 27.19 (26.22–28.15) | 1,607 | 24.44 (23.24–25.65) | 191 | 25.06 (21.44–28.67) |
| 2020 | 53,544 | 48.62 (48.21–49.04) | 3,399 | 26.64 (25.73–27.55) | 4,142 | 34.47 (33.41–35.53) | 1,921 | 27.60 (26.35–28.84) | 232 | 27.69 (24.05–31.32) |
| 2021 | 49,590 | 47.63 (47.21–48.05) | 3,007 | 24.49 (23.60–25.40) | 3,783 | 31.59 (30.58–32.63) | 1,883 | 27.88 (26.63–29.18) | 212 | 27.77 (24.08–31.89) |
| 2022 | 50,770 | 45.68 (45.28–46.08) | 3,036 | 23.46 (22.62–24.33) | 3,893 | 30.76 (29.79–31.75) | 1,958 | 26.64 (25.46–27.85) | 208 | 24.85 (21.54–28.54) |
| 2023 | 49,680 | 44.67 (44.28–45.07) | 2,939 | 22.25 (21.44–23.09) | 3,746 | 28.48 (27.57–29.43) | 1,935 | 25.20 (24.09–26.36) | 175 | 19.82 (16.96–23.05) |
| 2024 | 50,191 | 43.76 (43.38–44.15) | 3,033 | 21.71 (20.93–22.51) | 3,666 | 25.98 (25.14–26.85) | 1,959 | 23.53 (22.50–24.60) | 196 | 20.66 (17.85–23.83) |
| 2025 | 52,634 | 45.91 (45.51–46.30) | 3,224 | 23.24 (22.43–24.07) | 4,038 | 28.61 (27.73–29.52) | 2,106 | 25.29 (24.22–26.40) | 206 | 21.69 (18.81–24.93) |
| Total/Average | 1,006,092 | 38.07 (37.68–38.46) | 52,486 | 18.91 (18.04–19.78) | 59,467 | 25.50 (24.35–26.66) | 28,194 | 21.94 (20.47–23.42) | 3,641 | 23.94 (19.72–28.48) |

Race and ethnicity categories follow CDC WONDER classifications. Estimates for smaller groups, including American Indian or Alaska Native individuals, are based on smaller annual counts and should be interpreted cautiously. AAMRs are age-adjusted to the 2000 U.S. standard population and expressed per 100,000. Hispanic or Latino counts derive from a direct query on the Hispanic Origin field; because that extraction post-dates the race extraction, the five strata exceed the analytic total by 149 deaths (0.013%). The excess is confined to the single-race years: +28, +23, +25, +27 and +46 deaths in 2021 through 2025 respectively, and zero in every year from 1999 to 2020. Because it is an excess it cannot arise from the exclusion of unstated-origin or multiple-race records, which could only produce a deficit. The race counts in this table are from the 26 June 2026 extraction and the Hispanic-origin series from the direct query of 8 August 2026 (Methods 2.1).

**Supplementary Table S7.** Hispanic-origin distribution of Parkinson’s disease-related deaths, U.S. adults aged ≥45 years, 1999–2025, from direct query.

| Year | Hispanic or Latino, deaths | Hispanic AAMR | Not Hispanic or Latino, deathsᵃ | Hispanic origin not stated, deaths |
| --- | --- | --- | --- | --- |
| 1999 | 846 | 22.28 | 30,164 | 66 |
| 2000 | 918 | 22.82 | 31,375 | 77 |
| 2001 | 1,063 | 24.61 | 31,808 | 75 |
| 2002 | 1,020 | 22.50 | 32,507 | 82 |
| 2003 | 1,092 | 23.13 | 33,261 | 73 |
| 2004 | 1,140 | 22.71 | 32,576 | 44 |
| 2005 | 1,371 | 25.84 | 34,490 | 26 |
| 2006 | 1,370 | 24.73 | 32,895 | 38 |
| 2007 | 1,364 | 23.09 | 33,079 | 25 |
| 2008 | 1,471 | 23.83 | 32,841 | 43 |
| 2009 | 1,445 | 22.17 | 32,321 | 37 |
| 2010 | 1,639 | 24.27 | 33,536 | 36 |
| 2011 | 1,709 | 23.20 | 34,743 | 41 |
| 2012 | 1,803 | 23.24 | 35,100 | 77 |
| 2013 | 2,070 | 24.91 | 36,347 | 59 |
| 2014 | 2,101 | 23.73 | 36,784 | 63 |
| 2015 | 2,394 | 25.37 | 39,039 | 98 |
| 2016 | 2,526 | 25.50 | 41,239 | 90 |
| 2017 | 2,761 | 26.36 | 43,948 | 84 |
| 2018 | 2,982 | 27.20 | 46,317 | 87 |
| 2019 | 3,114 | 27.19 | 48,005 | 75 |
| 2020 | 4,142 | 34.47 | 59,096 | 104 |
| 2021 | 3,783 | 31.59 | 54,857 | 78 |
| 2022 | 3,893 | 30.76 | 56,136 | 100 |
| 2023 | 3,746 | 28.48 | 54,903 | 98 |
| 2024 | 3,666 | 25.98 | 55,556 | 112 |
| 2025 | 4,038 | 28.61 | 58,451 | 122 |

AAMR, age-adjusted mortality rate per 100,000 (2000 U.S. standard population). ᵃThis column is constructed on two different bases and is not a single continuous series. For 1999–2020 it is the annual total minus Hispanic or Latino deaths, and it therefore contains the deaths of unstated Hispanic origin shown in the adjacent column (1,400 across those 22 years); for that era it should be read as “not recorded as Hispanic or Latino” rather than as “recorded as not Hispanic or Latino”. For 2021–2025 it is the sum of the six single-race non-Hispanic groups, including “more than one race”, and therefore excludes unstated origin. The 2025 figure additionally derives from the 7 August 2026 re-query of the provisional single-race file, whereas Supplementary Table S6 reports the 26 June 2026 counts; the two differ by 102 deaths in that year alone (Methods 2.1).

**Supplementary Table S8.** Single-race detail underlying the combined Asian or Pacific Islander category, non-Hispanic, U.S. adults aged ≥45 years, 2018–2025.

| Year | Asian AAMR | Asian deaths | NHOPI AAMR | NHOPI deaths | More than one race, deaths |
| --- | --- | --- | --- | --- | --- |
| 2018 | 23.71 | 1,400 | 15.09 | 22 | 120 |
| 2019 | 24.71 | 1,541 | 20.95 | 29 | 131 |
| 2020 | 27.75 | 1,833 | 26.73 | 42 | 153 |
| 2021 | 27.98 | 1,848 | 22.07 | 35 | 165 |
| 2022 | 26.85 | 1,926 | 17.24 | 32 | 164 |
| 2023 | 25.30 | 1,896 | 20.65 | 39 | 174 |
| 2024 | 23.77 | 1,934 | 12.24 | 25 | 177 |
| 2025 (provisional) | 25.56 | 2,078 | 18.24 | 38 | 179 |

AAMR, age-adjusted mortality rate per 100,000 (2000 U.S. standard); NHOPI, Native Hawaiian or Other Pacific Islander. For 2021–2025, Asian and NHOPI were summed to reconstruct the combined category used in the bridged-race era (Methods 2.2). Those sums reproduce the Asian or Pacific Islander counts in Supplementary Table S6 exactly in 2021 through 2024. In 2025 they exceed it by 10 deaths (2,078 + 38 = 2,116 against 2,106), because the single-race detail in this table derives from the 7 August 2026 re-query of the provisional file whereas Supplementary Table S6 reports the 26 June 2026 counts (Methods 2.1). The 2018–2020 rows are shown for context and are not expected to reconcile with Supplementary Table S6, whose entries for those years are bridged-race counts.

**Supplementary Table S9: Trends in Age-Adjusted Mortality Rates and Deaths from Parkinson’s Disease among United States Adults, stratified by Urbanization, 1999–2020.**

| **Year** | **Metropolitan** | **Metropolitan** | **Non-metropolitan** | **Non-metropolitan** |
| --- | --- | --- | --- | --- |
| **Year** | **Deaths** | **AAMR (95% CI)** | **Deaths** | **AAMR (95% CI)** |
| 1999 | 25,178 | 32.99 (32.58–33.40) | 5,832 | 31.71 (30.90–32.52) |
| 2000 | 26,097 | 33.74 (33.33–34.15) | 6,196 | 33.45 (32.61–34.28) |
| 2001 | 26,667 | 33.83 (33.43–34.24) | 6,204 | 33.27 (32.44–34.10) |
| 2002 | 27,161 | 34.01 (33.60–34.41) | 6,366 | 33.94 (33.11–34.78) |
| 2003 | 27,843 | 34.27 (33.87–34.68) | 6,510 | 34.47 (33.63–35.31) |
| 2004 | 27,467 | 33.35 (32.95–33.74) | 6,249 | 32.88 (32.07–33.70) |
| 2005 | 29,173 | 34.73 (34.33–35.12) | 6,688 | 34.86 (34.03–35.70) |
| 2006 | 27,862 | 32.55 (32.17–32.93) | 6,403 | 32.92 (32.11–33.72) |
| 2007 | 27,972 | 32.06 (31.68–32.43) | 6,471 | 32.92 (32.11–33.72) |
| 2008 | 27,877 | 31.34 (30.97–31.71) | 6,435 | 32.48 (31.68–33.27) |
| 2009 | 27,487 | 30.33 (29.97–30.69) | 6,279 | 31.42 (30.64–32.20) |
| 2010 | 28,766 | 31.32 (30.96–31.69) | 6,409 | 31.68 (30.90–32.46) |
| 2011 | 29,925 | 31.61 (31.25–31.97) | 6,527 | 31.69 (30.92–32.46) |
| 2012 | 30,231 | 31.12 (30.76–31.47) | 6,672 | 31.92 (31.15–32.69) |
| 2013 | 31,654 | 31.79 (31.43–32.14) | 6,763 | 31.83 (31.07–32.60) |
| 2014 | 32,141 | 31.53 (31.18–31.88) | 6,744 | 31.31 (30.56–32.06) |
| 2015 | 34,221 | 32.81 (32.46–33.16) | 7,212 | 32.85 (32.09–33.62) |
| 2016 | 36,234 | 33.87 (33.52–34.22) | 7,531 | 33.91 (33.14–34.68) |
| 2017 | 38,442 | 35.02 (34.67–35.38) | 8,267 | 36.41 (35.62–37.20) |
| 2018 | 40,599 | 35.89 (35.54–36.24) | 8,700 | 37.52 (36.73–38.32) |
| 2019 | 42,019 | 36.23 (35.88–36.58) | 9,100 | 38.62 (37.82–39.41) |
| 2020 | 52,120 | 43.93 (43.55–44.31) | 11,118 | 46.43 (45.57–47.30) |
| Total/Average | 697,136 | 33.56 (33.19–33.93) | 154,676 | 34.02 (33.22–34.82) |

Urbanization is based on the 2013 NCHS urban-rural classification; observed urbanization data extend through 2020.

**Supplementary Table S10.** Six-level NCHS urbanization gradient in Parkinson’s disease-related mortality, U.S. adults aged ≥45 years, 1999–2020.

| Urbanization level | AAMR 1999 | AAMR 2020 | AAPC 1999–2020, % (95% CI); p | Segment APCs, % (95% CI); p |
| --- | --- | --- | --- | --- |
| Large Central Metro | 31.02 | 41.01 | +0.99 (+0.12 to +1.86); 0.025 | 1999–2012: −0.87 (−1.35 to −0.38); p=0.002. 2012–2018: +2.13 (+0.21 to +4.09); p=0.032. 2018–2020: +10.14 (+2.29 to +18.59); p=0.014 |
| Large Fringe Metro | 33.77 | 45.04 | +0.96 (+0.40 to +1.53); <0.001 | 1999–2015: −0.61 (−1.06 to −0.17); p=0.010. 2015–2020: +6.18 (+3.97 to +8.43); p<0.001 |
| Medium Metro | 34.03 | 45.13 | +0.80 (+0.15 to +1.45); 0.015 | 1999–2014: −0.71 (−1.30 to −0.13); p=0.020. 2014–2020: +4.69 (+2.69 to +6.74); p<0.001 |
| Small Metro | 34.96 | 46.64 | +1.08 (+0.61 to +1.54); <0.001 | 1999–2015: −0.37 (−0.73 to −0.01); p=0.045. 2015–2020: +5.85 (+4.03 to +7.70); p<0.001 |
| Micropolitan (Nonmetro) | 33.17 | 47.96 | +1.17 (+0.59 to +1.75); <0.001 | 1999–2015: −0.57 (−1.01 to −0.13); p=0.014. 2015–2020: +6.94 (+4.65 to +9.29); p<0.001 |
| NonCore (Nonmetro) | 30.02 | 44.53 | +1.42 (+0.88 to +1.96); <0.001 | 1999–2015: −0.20 (−0.61 to +0.20); p=0.305. 2015–2020: +6.79 (+4.67 to +8.96); p<0.001 |

AAMR, age-adjusted mortality rate per 100,000 (2000 U.S. standard population); AAPC, average annual percent change; APC, annual percent change. Every segment APC is now reported with its 95% confidence interval and p-value, and the asterisk convention used previously has been withdrawn because it was applied inconsistently. All thirteen segments differ significantly from zero at α=0.05 except the 1999–2015 decline in noncore counties (p=0.305). In particular the 2015–2020 segments in large fringe metropolitan (+6.18), small metropolitan (+5.85), micropolitan (+6.94) and noncore (+6.79) counties are all significant at p<0.001, consistent with the collapsed two-level non-metropolitan segment for the same years (+6.87; p<0.001, Supplementary Table S3). Urbanization follows the 2013 NCHS Urban–Rural Classification Scheme for Counties. The series ends in 2020 because the classification variable is not available thereafter.

**Supplementary Table S11: Trends in Age-Adjusted Mortality Rates and Deaths from Parkinson’s Disease among United States Adults, stratified by Census Region, 1999–2025.**

| **Year** | **Northeast** | **Northeast** | **Midwest** | **Midwest** | **South** | **South** | **West** | **West** |
| --- | --- | --- | --- | --- | --- | --- | --- | --- |
| **Year** | **Deaths** | **AAMR (95% CI)** | **Deaths** | **AAMR (95% CI)** | **Deaths** | **AAMR (95% CI)** | **Deaths** | **AAMR (95% CI)** |
| 1999 | 6,166 | 30.19 (29.44–30.95) | 8,387 | 36.46 (35.68–37.24) | 9,705 | 29.55 (28.96–30.14) | 6,752 | 36.73 (35.85–37.60) |
| 2000 | 6,529 | 31.49 (30.72–32.25) | 8,541 | 36.79 (36.01–37.57) | 10,344 | 31.11 (30.51–31.71) | 6,879 | 36.66 (35.80–37.53) |
| 2001 | 6,681 | 31.80 (31.04–32.56) | 8,598 | 36.63 (35.86–37.41) | 10,422 | 30.90 (30.30–31.49) | 7,170 | 37.20 (36.34–38.06) |
| 2002 | 6,672 | 31.43 (30.67–32.18) | 8,967 | 37.84 (37.06–38.63) | 10,655 | 31.26 (30.67–31.85) | 7,233 | 36.86 (36.01–37.71) |
| 2003 | 6,574 | 30.59 (29.85–31.33) | 8,954 | 37.29 (36.52–38.07) | 11,177 | 32.27 (31.67–32.87) | 7,648 | 38.08 (37.23–38.94) |
| 2004 | 6,527 | 30.18 (29.45–30.91) | 8,843 | 36.53 (35.77–37.29) | 10,892 | 31.00 (30.42–31.59) | 7,454 | 36.48 (35.66–37.31) |
| 2005 | 6,954 | 31.74 (30.99–32.49) | 9,464 | 38.64 (37.86–39.42) | 11,654 | 32.44 (31.85–33.03) | 7,789 | 37.06 (36.23–37.88) |
| 2006 | 6,493 | 29.32 (28.60–30.03) | 8,841 | 35.57 (34.83–36.32) | 11,188 | 30.54 (29.97–31.10) | 7,743 | 35.99 (35.19–36.79) |
| 2007 | 6,648 | 29.71 (28.99–30.43) | 8,746 | 34.75 (34.02–35.48) | 11,518 | 30.81 (30.25–31.37) | 7,531 | 34.25 (33.48–35.03) |
| 2008 | 6,454 | 28.52 (27.82–29.22) | 8,858 | 34.82 (34.09–35.54) | 11,284 | 29.53 (28.98–30.07) | 7,716 | 34.24 (33.48–35.01) |
| 2009 | 6,380 | 27.90 (27.21–28.59) | 8,630 | 33.58 (32.87–34.29) | 11,208 | 28.75 (28.22–29.29) | 7,548 | 32.65 (31.91–33.39) |
| 2010 | 6,580 | 28.63 (27.93–29.33) | 8,803 | 33.88 (33.16–34.59) | 11,928 | 30.06 (29.52–30.60) | 7,864 | 33.40 (32.66–34.14) |
| 2011 | 6,873 | 29.42 (28.71–30.12) | 9,018 | 34.14 (33.43–34.85) | 12,224 | 29.80 (29.27–30.33) | 8,337 | 34.08 (33.34–34.81) |
| 2012 | 6,622 | 27.81 (27.13–28.48) | 9,263 | 34.50 (33.79–35.21) | 12,596 | 29.86 (29.34–30.39) | 8,422 | 33.49 (32.76–34.21) |
| 2013 | 7,144 | 29.63 (28.93–30.33) | 9,528 | 34.90 (34.19–35.61) | 13,166 | 30.23 (29.71–30.75) | 8,579 | 33.13 (32.42–33.84) |
| 2014 | 7,057 | 28.86 (28.18–29.54) | 9,672 | 34.95 (34.25–35.66) | 13,415 | 29.99 (29.48–30.50) | 8,741 | 32.68 (31.98–33.37) |
| 2015 | 7,301 | 29.64 (28.95–30.34) | 9,822 | 34.90 (34.20–35.60) | 14,690 | 31.87 (31.36–32.39) | 9,620 | 34.85 (34.15–35.55) |
| 2016 | 7,578 | 30.41 (29.72–31.11) | 10,380 | 36.45 (35.74–37.16) | 15,786 | 33.23 (32.71–33.75) | 10,021 | 35.36 (34.66–36.06) |
| 2017 | 7,964 | 30.99 (30.30–31.68) | 10,926 | 37.55 (36.84–38.26) | 17,269 | 35.35 (34.82–35.88) | 10,550 | 36.35 (35.65–37.05) |
| 2018 | 8,431 | 32.28 (31.58–32.97) | 11,590 | 39.00 (38.28–39.71) | 18,237 | 36.10 (35.58–36.63) | 11,041 | 36.80 (36.11–37.50) |
| 2019 | 8,406 | 31.73 (31.05–32.42) | 11,978 | 39.63 (38.91–40.34) | 19,088 | 36.63 (36.11–37.15) | 11,647 | 37.73 (37.04–38.42) |
| 2020 | 10,880 | 40.56 (39.79–41.33) | 15,077 | 49.37 (48.58–50.17) | 23,713 | 44.29 (43.72–44.85) | 13,568 | 42.79 (42.07–43.52) |
| 2021 | 9,456 | 36.63 (35.89–37.39) | 13,235 | 45.68 (44.90–46.47) | 22,584 | 44.46 (43.88–45.05) | 13,172 | 44.23 (43.47–44.99) |
| 2022 | 9,475 | 34.76 (34.06–35.47) | 13,603 | 44.18 (43.43–44.93) | 23,185 | 42.55 (42.00–43.10) | 13,579 | 42.33 (41.61–43.05) |
| 2023 | 9,347 | 34.22 (33.53–34.93) | 13,267 | 43.17 (42.43–43.91) | 22,905 | 41.61 (41.07–42.16) | 12,931 | 39.87 (39.18–40.57) |
| 2024 | 9,534 | 33.62 (32.95–34.31) | 13,394 | 41.97 (41.26–42.70) | 23,053 | 40.14 (39.62–40.66) | 13,037 | 38.51 (37.85–39.18) |
| 2025 | 10,027 | 35.38 (34.69–36.08) | 14,140 | 44.39 (43.66–45.13) | 24,548 | 42.72 (42.19–43.27) | 13,447 | 39.71 (39.04–40.39) |
| Total/Average | 204,753 | 31.39 (30.67–32.10) | 280,525 | 38.06 (37.32–38.80) | 408,434 | 33.96 (33.41–34.52) | 256,019 | 36.72 (35.97–37.48) |

AAMR, age-adjusted mortality rate per 100,000 (2000 U.S. standard). Deaths were identified by ICD-10 code G20 recorded anywhere on the death certificate.

**Supplementary Table S12: Age-adjusted mortality rates and deaths from Parkinson’s Disease among United States Adults, stratified by State, 1999–2020 and 2021–2025.**

| **State** | **Deaths (1999–2020)** | **AAMR, 95% CI (1999–2020)** | **Deaths (2021–2025)** | **AAMR, 95% CI (2021–2025)** | **Deaths (1999–2025)** |
| --- | --- | --- | --- | --- | --- |
| Alabama | 12,269 | 31.52 (30.96–32.08) | 4,612 | 40.85 (39.67–42.06) | 16,881 |
| Alaska | 897 | 31.92 (29.78–34.07) | 374 | 35.45 (31.82–39.40) | 1,271 |
| Arizona | 17,129 | 32.37 (31.89–32.86) | 6,722 | 37.29 (36.40–38.20) | 23,851 |
| Arkansas | 7,590 | 30.24 (29.56–30.93) | 2,871 | 42.30 (40.76–43.89) | 10,461 |
| California | 97,082 | 35.97 (35.74–36.20) | 31,104 | 39.56 (39.12–40.01) | 128,186 |
| Colorado | 12,487 | 37.77 (37.10–38.43) | 5,197 | 47.94 (46.63–49.27) | 17,684 |
| Connecticut | 10,422 | 30.57 (29.97–31.16) | 3,071 | 34.90 (33.67–36.17) | 13,493 |
| Delaware | 2,343 | 30.54 (29.30–31.78) | 1,075 | 41.92 (39.43–44.56) | 3,418 |
| District of Columbia | 1,109 | 24.62 (23.16–26.08) | 370 | 32.20 (28.99–35.70) | 1,479 |
| Florida | 58,249 | 27.91 (27.68–28.14) | 23,884 | 36.79 (36.33–37.26) | 82,133 |
| Georgia | 17,933 | 30.08 (29.64–30.52) | 7,725 | 38.78 (37.91–39.67) | 25,658 |
| Hawaii | 3,681 | 28.71 (27.78–29.65) | 994 | 26.42 (24.78–28.17) | 4,675 |
| Idaho | 4,653 | 39.90 (38.75–41.05) | 1,865 | 47.47 (45.32–49.70) | 6,518 |
| Illinois | 35,650 | 34.49 (34.13–34.85) | 10,617 | 38.48 (37.75–39.22) | 46,267 |
| Indiana | 19,592 | 37.49 (36.96–38.02) | 6,635 | 46.24 (45.13–47.37) | 26,227 |
| Iowa | 11,747 | 38.43 (37.73–39.13) | 3,666 | 48.19 (46.63–49.79) | 15,413 |
| Kansas | 9,602 | 38.32 (37.55–39.09) | 3,055 | 47.62 (45.94–49.36) | 12,657 |
| Kentucky | 12,242 | 35.74 (35.10–36.37) | 4,468 | 46.44 (45.08–47.84) | 16,710 |
| Louisiana | 10,333 | 30.51 (29.92–31.10) | 3,786 | 39.93 (38.66–41.24) | 14,119 |
| Maine | 4,755 | 36.20 (35.17–37.23) | 1,575 | 40.99 (38.98–43.10) | 6,330 |
| Maryland | 15,350 | 34.97 (34.41–35.52) | 5,567 | 42.19 (41.09–43.33) | 20,917 |
| Massachusetts | 17,719 | 29.34 (28.90–29.78) | 5,832 | 36.06 (35.14–37.01) | 23,551 |
| Michigan | 29,557 | 34.74 (34.34–35.13) | 9,483 | 41.16 (40.33–42.00) | 39,040 |
| Minnesota | 18,715 | 41.60 (40.99–42.20) | 6,397 | 50.25 (49.02–51.51) | 25,112 |
| Mississippi | 7,371 | 32.43 (31.69–33.17) | 3,033 | 48.54 (46.81–50.32) | 10,404 |
| Missouri | 18,390 | 35.20 (34.69–35.71) | 6,121 | 43.55 (42.46–44.66) | 24,511 |
| Montana | 3,059 | 33.76 (32.56–34.96) | 1,095 | 40.81 (38.41–43.34) | 4,154 |
| Nebraska | 7,316 | 44.32 (43.29–45.34) | 2,300 | 54.30 (52.09–56.59) | 9,616 |
| Nevada | 4,715 | 26.66 (25.89–27.43) | 2,222 | 34.54 (33.10–36.03) | 6,937 |
| New Hampshire | 3,966 | 35.64 (34.52–36.76) | 1,480 | 42.88 (40.71–45.16) | 5,446 |
| New Jersey | 23,627 | 30.79 (30.40–31.19) | 6,754 | 32.24 (31.47–33.02) | 30,381 |
| New Mexico | 5,514 | 34.58 (33.66–35.49) | 1,857 | 36.93 (35.25–38.67) | 7,371 |
| New York | 44,927 | 26.35 (26.11–26.60) | 14,055 | 29.95 (29.46–30.45) | 58,982 |
| North Carolina | 23,874 | 32.77 (32.35–33.19) | 9,302 | 40.91 (40.08–41.76) | 33,176 |
| North Dakota | 2,490 | 36.79 (35.33–38.26) | 683 | 39.49 (36.55–42.64) | 3,173 |
| Ohio | 38,375 | 37.47 (37.09–37.84) | 11,691 | 43.42 (42.64–44.22) | 50,066 |
| Oklahoma | 11,305 | 36.99 (36.31–37.67) | 4,414 | 54.27 (52.67–55.91) | 15,719 |
| Oregon | 13,143 | 39.13 (38.46–39.80) | 4,810 | 49.00 (47.61–50.42) | 17,953 |
| Pennsylvania | 45,442 | 35.05 (34.73–35.38) | 13,128 | 40.14 (39.45–40.84) | 58,570 |
| Rhode Island | 3,617 | 34.30 (33.17–35.43) | 1,101 | 41.51 (39.08–44.07) | 4,718 |
| South Carolina | 11,976 | 32.94 (32.35–33.53) | 5,386 | 44.39 (43.20–45.61) | 17,362 |
| South Dakota | 2,866 | 36.42 (35.07–37.76) | 905 | 45.14 (42.22–48.24) | 3,771 |
| Tennessee | 17,522 | 35.05 (34.53–35.57) | 7,024 | 47.35 (46.24–48.48) | 24,546 |
| Texas | 57,622 | 37.20 (36.90–37.51) | 22,768 | 46.32 (45.71–46.93) | 80,390 |
| Utah | 6,208 | 41.54 (40.50–42.57) | 2,355 | 48.58 (46.62–50.61) | 8,563 |
| Vermont | 2,439 | 43.03 (41.32–44.75) | 843 | 50.19 (46.84–53.77) | 3,282 |
| Virginia | 18,895 | 32.29 (31.83–32.75) | 7,759 | 42.60 (41.65–43.56) | 26,654 |
| Washington | 20,051 | 39.04 (38.50–39.58) | 7,112 | 45.21 (44.16–46.28) | 27,163 |
| West Virginia | 6,176 | 34.70 (33.83–35.57) | 2,231 | 48.35 (46.35–50.43) | 8,407 |
| Wisconsin | 18,586 | 36.69 (36.16–37.22) | 6,086 | 44.76 (43.63–45.91) | 24,672 |
| Wyoming | 1,234 | 29.43 (27.78–31.08) | 459 | 36.26 (32.99–39.81) | 1,693 |
| Total (50 states + DC) | 851,812 | — | 297,919 | — | 1,149,731 |

AAMR, age-adjusted mortality rate per 100,000 (2000 U.S. standard). Values are pooled period rates and total deaths for each window; the final column is total deaths for 1999–2025. CDC WONDER flags rates based on fewer than 20 deaths as unreliable.

**Supplementary Table S13.** Ensemble composition, selected ARIMA order, and rolling-origin back-test performance by forecast horizon, by stratum.

| Stratum | Weight: Holt | Weight: damped | Weight: ARIMA | ARIMA order | MAPE h=1 | MAPE h=3 | MAPE h=5 | MAPE h=10 | Bias h=5, %: primary; pre-pandemic | Projected |
| --- | --- | --- | --- | --- | --- | --- | --- | --- | --- | --- |
| Overall | 0.262 | 0.253 | 0.486 | (0,1,0) trend=n | 4.2% | 9.8% | 13.4% | 24.0% | -6.4; -11.4 | Yes |
| Female | 0.241 | 0.248 | 0.511 | (0,1,0) trend=n | 4.3% | 10.2% | 13.3% | 23.3% | -6.4; -9.6 | Yes |
| Male | 0.233 | 0.256 | 0.510 | (0,1,0) trend=n | 4.3% | 10.2% | 13.0% | 23.2% | -6.0; -11.0 | Yes |
| 45–64 | 0.297 | 0.279 | 0.424 | (0,1,0) trend=n | 5.6% | 11.5% | 17.2% | 31.2% | -12.8; -16.0 | Yes |
| ≥65 | 0.269 | 0.240 | 0.492 | (0,1,0) trend=n | 4.3% | 9.6% | 13.8% | 23.8% | -6.5; -11.3 | Yes |
| White, non-Hispanic | 0.296 | 0.250 | 0.454 | (0,1,0) trend=n | 4.0% | 9.1% | 12.9% | 25.7% | -8.7; -11.9 | Yes |
| Hispanic or Latino | 0.336 | 0.328 | 0.337 | (1,1,0) trend=n | 5.6% | 9.0% | 8.6% | 14.4% | -6.9; -9.1 | Yes |
| American Indian or Alaska Native, non-Hispanic | 0.309 | 0.321 | 0.370 | (0,0,1) trend=c | 8.0% | 11.8% | 11.2% | 7.6% | +0.3; -7.5 | Yes |
| Asian or Pacific Islander, non-Hispanic | 0.298 | 0.251 | 0.451 | (1,1,1) trend=n | 7.7% | 11.9% | 16.6% | 26.0% | -9.4; -16.2 | Yes |
| Black or African American, non-Hispanic | 0.275 | 0.286 | 0.439 | (0,1,0) trend=n | 6.1% | 13.5% | 16.3% | 27.6% | -9.6; -17.5 | Yes |
| Northeast | 0.313 | 0.263 | 0.424 | (1,0,0) trend=c | 4.5% | 8.7% | 11.2% | 19.0% | -4.8; -8.0 | Yes |
| Midwest | 0.243 | 0.228 | 0.529 | (0,1,0) trend=n | 3.9% | 9.7% | 14.6% | 22.8% | -4.3; -10.1 | Yes |
| South | 0.327 | 0.324 | 0.349 | (0,1,0) trend=t | 5.3% | 11.0% | 14.6% | 29.5% | -9.9; -14.1 | Yes |
| West | 0.339 | 0.327 | 0.333 | (0,0,3) trend=c | 6.8% | 10.5% | 14.2% | 20.4% | -8.7; -10.3 | Yes |
| Metropolitan | 0.297 | 0.325 | 0.378 | (0,1,0) trend=t | 4.0% | 8.4% | 13.7% | — | -13.7; -11.4 | No |
| Non-metropolitan | 0.334 | 0.341 | 0.325 | (1,1,2) trend=t | 5.1% | 8.7% | 13.1% | — | -13.1; -11.6 | No |

MAPE, mean absolute percentage error from rolling-origin back-testing on the log scale, expressed on the rate scale; h, forecast horizon in years. Weights, ARIMA order and MAPE are for the primary specification fitted to the full 1999–2025 series. Weights are inversely proportional to mean back-test error over horizons of one to five years and are non-negative; they are displayed to three decimal places and may therefore sum to 0.999 or 1.001, whereas the fitted weights sum to one by construction. Bias is the mean signed percentage error at the five-year horizon, negative values indicating under-prediction; the first figure is for the primary specification and the second, after the semicolon, for the pre-pandemic-anchored sensitivity specification fitted to 1999–2019. The pre-pandemic anchor is negatively biased in all 16 strata, so the two specifications under-predict in the same direction rather than in opposite directions (Results 3.8). Back-testing used rolling origins with a minimum training window of 12 years, and the pandemic years 2020 and 2021 were retained in training but excluded as back-test targets; this is stated in Methods 2.5, and it makes every error figure in this table more favorable than it would be if those years were scored. In the metropolitan and non-metropolitan strata the five-year bias equals the five-year MAPE in magnitude because every back-test error at that horizon carried the same sign. Those two series end in 2020, so with the pandemic years excluded the only available five-year targets are 2015 through 2019, all of which fall inside the steep late-period rise and are under-predicted from every origin; this is a property of the series, not a transcription error. Model selection is described in Methods 2.5.

**Supplementary Table S14.** Independent replication: 2030 projections from a separately implemented ARIMA analysis compared with the primary ensemble.

| Stratum | Observed 2025 | Independent ARIMA, 2030 | Random walk selected | Primary ensemble, 2030 (95% PI) | Difference |
| --- | --- | --- | --- | --- | --- |
| Overall | 41.03 | 41.03 | Yes | 41.73 (33.97 to 51.27) | +1.7% |
| Female | 26.37 | 26.37 | Yes | 26.52 (21.30 to 33.01) | +0.6% |
| Male | 61.01 | 61.01 | Yes | 61.53 (50.37 to 75.16) | +0.9% |
| 45–64 years | 1.52 | 1.52 | Yes | 1.54 (1.12 to 2.14) | +1.6% |
| ≥65 years | 110.45 | 110.45 | Yes | 112.27 (91.40 to 137.91) | +1.7% |
| White, non-Hispanic | 45.91 | 45.91 | Yes | 47.00 (38.30 to 57.68) | +2.4% |
| Black or African American, non-Hispanic | 23.24 | 23.24 | Yes | 23.50 (17.68 to 31.24) | +1.1% |
| Hispanic or Latino | 28.61 | 28.17 | No | 29.28 (23.43 to 36.60) | +4.0% |
| Asian or Pacific Islander, non-Hispanic | 25.29 | 24.74 | No | 25.17 (19.67 to 32.21) | +1.7% |
| American Indian or Alaska Native, non-Hispanic | 21.69 | 23.93 | No | 23.47 (19.28 to 28.56) | -1.9% |
| Northeast | 35.38 | 34.88 | No | 34.08 (27.93 to 41.59) | -2.3% |
| Midwest | 44.39 | 44.39 | Yes | 44.85 (35.86 to 56.09) | +1.0% |
| South | 42.72 | 42.72 | Yes | 46.74 (37.11 to 58.86) | +9.4% |
| West | 39.71 | 39.71 | Yes | 38.96 (32.63 to 46.51) | -1.9% |

PI, prediction interval. Point estimates only are compared; the independent implementation generated intervals on the rate scale (Methods 2.5). Urbanization is excluded because those strata are not projected. Percentage differences are computed on the unrounded projections and may therefore differ slightly from a recalculation using the two-decimal values displayed; the largest such case is the 45–64 stratum, shown as +1.6% here against +1.3% if recomputed from the rounded figures.

**Supplementary Table S15.** COVID-19 (U07.1) co-listing among Parkinson’s disease-mentioned deaths, U.S. adults aged ≥45 years, 2019–2025.

| Year | PD-mentioned deaths | Also listing U07.1 | % of PD-mentioned deaths | PD AAMR | AAMR, PD with U07.1 | AAMR, PD excluding U07.1 |
| --- | --- | --- | --- | --- | --- | --- |
| 2019 | 51,119 | 0 | 0.00% | 36.63 | 0.00 | 36.63 |
| 2020 | 63,238 | 6,421 | 10.15% | 44.36 | 4.48 | 39.88 |
| 2021 | 58,447 | 4,192 | 7.17% | 43.13 | 3.05 | 40.08 |
| 2022 | 59,842 | 3,701 | 6.18% | 41.40 | 2.52 | 38.88 |
| 2023 | 58,450 | 1,769 | 3.03% | 40.16 | 1.21 | 38.95 |
| 2024 | 59,018 | 1,086 | 1.84% | 38.93 | 0.73 | 38.20 |
| 2025 (provisional) | 62,162 | 488 | 0.79% | 41.03 | 0.32 | 40.71 |

AAMR, age-adjusted mortality rate per 100,000, standardized to the 2000 U.S. standard population.

**Supplementary Table S16.** Projected Parkinson’s disease-related death counts among U.S. adults aged ≥45 years, 2026–2030, with a constant-rate scenario isolating the demographic contribution.

| Year | Projected deaths, 45–64 | Projected deaths, ≥65 | Projected deaths, total (95% PI) | Constant-rate scenario, total | Projected population ≥65 |
| --- | --- | --- | --- | --- | --- |
| 2025 (observed) | 1,535 | 60,627 | 62,162 | 62,162 | 63,326,603 |
| 2026 | 1,537 | 62,488 | 64,025 (58,316 to 70,294) | 63,862 | 65,106,632 |
| 2027 | 1,537 | 64,337 | 65,874 (57,781 to 75,107) | 65,479 | 66,798,846 |
| 2028 | 1,540 | 66,092 | 67,632 (57,601 to 79,421) | 66,994 | 68,382,810 |
| 2029 | 1,543 | 67,767 | 69,310 (57,563 to 83,471) | 68,420 | 69,873,988 |
| 2030 | 1,551 | 69,274 | 70,825 (57,517 to 87,237) | 69,675 | 71,183,498 |

PI, prediction interval. Counts were obtained by scaling the observed 2025 band-specific death rate by the ratio of the projected to the observed 2025 age-adjusted rate for that band, and applying the result to the projected population.

**Supplementary Table S17.** Ascertainment sensitivity: Parkinson’s disease as underlying cause versus any mention, U.S. adults aged ≥45 years, 1999–2025.

| Year | Any-mention deaths | Underlying-cause deaths | Underlying cause, % | Mentioned but not underlying | Any-mention AAMR | Underlying-cause AAMR | Contributing-only AAMR |
| --- | --- | --- | --- | --- | --- | --- | --- |
| 1999 | 31,010 | 14,478 | 46.7% | 16,532 | 32.74 | 15.28 | 17.46 |
| 2000 | 32,293 | 15,549 | 48.1% | 16,744 | 33.66 | 16.21 | 17.45 |
| 2001 | 32,871 | 16,412 | 49.9% | 16,459 | 33.73 | 16.84 | 16.89 |
| 2002 | 33,527 | 16,819 | 50.2% | 16,708 | 34.01 | 17.03 | 16.98 |
| 2003 | 34,353 | 17,846 | 51.9% | 16,507 | 34.29 | 17.80 | 16.49 |
| 2004 | 33,716 | 17,864 | 53.0% | 15,852 | 33.25 | 17.61 | 15.64 |
| 2005 | 35,861 | 19,406 | 54.1% | 16,455 | 34.75 | 18.81 | 15.94 |
| 2006 | 34,265 | 19,404 | 56.6% | 14,861 | 32.59 | 18.46 | 14.13 |
| 2007 | 34,443 | 19,893 | 57.8% | 14,550 | 32.21 | 18.61 | 13.60 |
| 2008 | 34,312 | 20,289 | 59.1% | 14,023 | 31.53 | 18.65 | 12.88 |
| 2009 | 33,766 | 20,368 | 60.3% | 13,398 | 30.52 | 18.45 | 12.07 |
| 2010 | 35,175 | 21,817 | 62.0% | 13,358 | 31.38 | 19.45 | 11.93 |
| 2011 | 36,452 | 22,949 | 63.0% | 13,503 | 31.63 | 19.94 | 11.69 |
| 2012 | 36,903 | 23,618 | 64.0% | 13,285 | 31.28 | 20.03 | 11.25 |
| 2013 | 38,417 | 24,973 | 65.0% | 13,444 | 31.81 | 20.69 | 11.12 |
| 2014 | 38,885 | 25,926 | 66.7% | 12,959 | 31.53 | 21.03 | 10.50 |
| 2015 | 41,433 | 27,723 | 66.9% | 13,710 | 32.81 | 21.95 | 10.86 |
| 2016 | 43,765 | 29,438 | 67.3% | 14,327 | 33.87 | 22.86 | 11.01 |
| 2017 | 46,709 | 31,688 | 67.8% | 15,021 | 35.25 | 23.97 | 11.28 |
| 2018 | 49,299 | 33,529 | 68.0% | 15,770 | 36.16 | 24.64 | 11.52 |
| 2019 | 51,119 | 35,025 | 68.5% | 16,094 | 36.63 | 25.13 | 11.50 |
| 2020 | 63,238 | 39,957 | 63.2% | 23,281 | 44.36 | 28.10 | 16.26 |
| 2021 | 58,447 | 38,108 | 65.2% | 20,339 | 43.13 | 28.23 | 14.90 |
| 2022 | 59,842 | 39,431 | 65.9% | 20,411 | 41.40 | 27.33 | 14.07 |
| 2023 | 58,450 | 39,714 | 67.9% | 18,736 | 40.16 | 27.35 | 12.81 |
| 2024 | 59,018 | 40,414 | 68.5% | 18,604 | 38.93 | 26.71 | 12.22 |
| 2025 | 62,162 | 43,020 | 69.2% | 19,142 | 41.03 | 28.45 | 12.58 |

AAMR, age-adjusted mortality rate per 100,000 (2000 U.S. standard population). The contributing-only AAMR is the difference between the any-mention and underlying-cause rates. The two components are mutually exclusive and share the same population denominator, so their age-adjusted rates are additive. Neither component is monotonic. The contributing-only rate fell from 17.46 per 100,000 in 1999 to a trough of 10.50 in 2014, recovered to 11.50 by 2019, rose to 16.26 in the pandemic year 2020, and stood at 12.58 in 2025 — a net fall of 27.9% across the window, against a rise of 86.2% in the underlying-cause rate. On counts rather than rates the same component fell from 16,532 deaths in 1999 to 12,959 in 2014 and then rose 47.7% to 19,142 in 2025, so the full-window count change of +15.8% reflects 1999 being a local high and should not be read as the behaviour of the series (Discussion 4.2).

**Supplementary Table S18.** Joinpoint trend estimates restricted to final data (1999–2024), compared with the primary analysis including the provisional year.

| Stratum | Analysis | AAPC, % (95% CI); p | Segment APCs, % |
| --- | --- | --- | --- |
| Overall | Primary, 1999–2025 | 0.56 (−0.10 to 1.22); 0.099 | 1999–2014: −0.65*; 2014–2021: +4.70*; 2021–2025: −1.96 |
| Overall | Final data only, 1999–2024 | +0.51 (-0.21 to +1.23); 0.163 | 1999–2014: -0.67*; 2014–2021: +4.88*; 2021–2024: -3.44 |
| Female | Primary, 1999–2025 | 0.33 (−0.25 to 0.92); 0.267 | 1999–2015: −0.87*; 2015–2020: +6.13*; 2020–2025: −1.41 |
| Female | Final data only, 1999–2024 | +0.22 (-0.58 to +1.03); 0.586 | 1999–2014: -1.03*; 2014–2021: +4.75*; 2021–2024: -3.74 |
| Male | Primary, 1999–2025 | 0.40 (−0.25 to 1.05); 0.225 | 1999–2014: −0.83*; 2014–2021: +4.51*; 2021–2025: −1.98 |
| Male | Final data only, 1999–2024 | +0.34 (-0.34 to +1.02); 0.329 | 1999–2014: -0.85*; 2014–2021: +4.69*; 2021–2024: -3.56 |

AAPC, average annual percent change; APC, annual percent change; asterisk denotes significance at α=0.05. Data for 2025 are provisional; data through 2024 are final. Joinpoint Regression Program v5.4.0, log-linear model, Monte Carlo permutation test, weighted Bayesian Information Criterion.

**Supplementary Table S19.** Case-definition sensitivity: G20 alone versus G20 with G21 (secondary parkinsonism), U.S. adults aged ≥45 years, 1999–2025.

| Year | G20 deaths | G20+G21 deaths | Added by G21 | G20 AAMR | G20+G21 AAMR | Difference |
| --- | --- | --- | --- | --- | --- | --- |
| 1999 | 31,010 | 31,107 | 97 (0.31%) | 32.74 | 32.84 | +0.10 |
| 2000 | 32,293 | 32,417 | 124 (0.38%) | 33.66 | 33.79 | +0.13 |
| 2001 | 32,871 | 33,010 | 139 (0.42%) | 33.73 | 33.86 | +0.13 |
| 2002 | 33,527 | 33,713 | 186 (0.55%) | 34.01 | 34.18 | +0.17 |
| 2003 | 34,353 | 34,530 | 177 (0.52%) | 34.29 | 34.50 | +0.21 |
| 2004 | 33,716 | 33,857 | 141 (0.42%) | 33.25 | 33.38 | +0.13 |
| 2005 | 35,861 | 36,048 | 187 (0.52%) | 34.75 | 34.93 | +0.18 |
| 2006 | 34,265 | 34,477 | 212 (0.62%) | 32.59 | 32.81 | +0.22 |
| 2007 | 34,443 | 34,712 | 269 (0.78%) | 32.21 | 32.49 | +0.28 |
| 2008 | 34,312 | 34,807 | 495 (1.44%) | 31.53 | 32.01 | +0.48 |
| 2009 | 33,766 | 34,309 | 543 (1.61%) | 30.52 | 31.04 | +0.52 |
| 2010 | 35,175 | 35,673 | 498 (1.42%) | 31.38 | 31.79 | +0.41 |
| 2011 | 36,452 | 36,965 | 513 (1.41%) | 31.63 | 32.05 | +0.42 |
| 2012 | 36,903 | 37,429 | 526 (1.43%) | 31.28 | 31.73 | +0.45 |
| 2013 | 38,417 | 38,924 | 507 (1.32%) | 31.81 | 32.22 | +0.41 |
| 2014 | 38,885 | 39,472 | 587 (1.51%) | 31.53 | 31.95 | +0.42 |
| 2015 | 41,433 | 42,041 | 608 (1.47%) | 32.81 | 33.26 | +0.45 |
| 2016 | 43,765 | 44,438 | 673 (1.54%) | 33.87 | 34.42 | +0.55 |
| 2017 | 46,709 | 47,370 | 661 (1.42%) | 35.25 | 35.74 | +0.49 |
| 2018 | 49,299 | 49,967 | 668 (1.35%) | 36.16 | 36.63 | +0.47 |
| 2019 | 51,119 | 51,845 | 726 (1.42%) | 36.63 | 37.14 | +0.51 |
| 2020 | 63,238 | 64,135 | 897 (1.42%) | 44.36 | 44.99 | +0.63 |
| 2021 | 58,447 | 59,276 | 829 (1.42%) | 43.13 | 43.73 | +0.60 |
| 2022 | 59,842 | 60,799 | 957 (1.60%) | 41.40 | 42.06 | +0.66 |
| 2023 | 58,450 | 59,304 | 854 (1.46%) | 40.16 | 40.74 | +0.58 |
| 2024 | 59,018 | 59,935 | 917 (1.55%) | 38.93 | 39.53 | +0.60 |
| 2025 | 62,162 | 63,187 | 1,025 (1.65%) | 41.03 | 41.71 | +0.68 |

AAMR, age-adjusted mortality rate per 100,000 (2000 U.S. standard population). Percentages in the “Added by G21” column are expressed as a proportion of G20 deaths in the same year, not of the combined G20+G21 total. The increment widens across the window, from +0.10 per 100,000 in 1999 to +0.68 in 2025, so adding G21 slightly steepens the trend rather than shifting the series by a constant amount (Results 3.11).
